# Low‐Intensity Pulsed Ultrasound Treatment Selectively Stimulates Senescent Cells to Promote SASP Factors for Immune Cell Recruitment

**DOI:** 10.1111/acel.14486

**Published:** 2025-01-16

**Authors:** HyeRan Gwak, Seoyoung Hong, Su Hyun Lee, In Woo Kim, Yonghan Kim, Hyungmin Kim, Ki Joo Pahk, So Yeon Kim

**Affiliations:** ^1^ Chemical and Biological Integrative Research Center, Biomedical Research Division Korea Institute of Science and Technology Seoul Republic of Korea; ^2^ Bionics Research Center, Biomedical Research Division Korea Institute of Science and Technology Seoul Republic of Korea; ^3^ Division of Bio‐Medical Science and Technology, KIST School Korea University of Science and Technology (UST) Seoul Republic of Korea; ^4^ KHU‐KIST Department of Converging Science and Technology Kyung Hee University Seoul Republic of Korea; ^5^ Department of Biomedical Engineering Kyung Hee University Yongin Republic of Korea

**Keywords:** LIPUS stimulation, macrophage recruitment, phagocytosis, senescence associated secretory phenotype, senescent cells

## Abstract

As emerging therapeutic strategies for aging and age‐associated diseases, various biochemical approaches have been developed to selectively remove senescent cells, but how physical stimulus influences senescent cells and its possible application in senolytic therapy has not been reported yet. Here we developed a physical method to selectively stimulate senescent cells via low‐intensity pulsed ultrasound (LIPUS) treatment. LIPUS stimulation did not affect the cell cycle, but selectively enhanced secretion of specific cytokines in senescent cells, known as the senescence‐associated secretory phenotype (SASP), resulting in enhanced migration of monocytes/macrophages and upregulation of phagocytosis of senescent cells by M1 macrophage. We found that LIPUS stimulation selectively perturbed the cellular membrane structure in senescent cells, which led to activation of the intracellular reactive oxygen species‐dependent p38‐NF‐κB signaling pathway. Using a UV‐induced skin aging mouse model, we confirmed enhanced macrophage infiltration followed by reduced senescent cells after LIPUS treatment. Due to the advantages of ultrasound treatment, such as non‐invasiveness, deep penetration capability, and easy application in clinical settings, we expect that our method can be applied to treat various senescence‐associated diseases or combined with other established biochemical therapies to enhance efficacy.

## Graphical abstract

The regulatory effects of low‐intensity pulsed ultrasound (LIPUS) stimulation in senescent cells are presented. LIPUS perturbs the membrane in senescent cells and induces intracellular reactive oxygen species via NOX4 expression. Consequently, secretion of senescence‐associated secretory phenotype, followed by migration of monocytes/macrophages and phagocytosis of senescent cells can be enhanced.

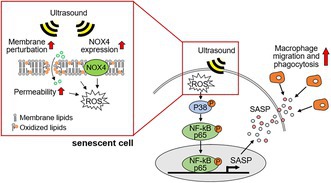

## 1 Introduction

Cellular senescence is a state of permanent cell cycle arrest in response to a variety of cellular stresses such as oxidative stress, DNA damage, and oncogene activation (see Hernandez‐Segura, Nehme, and Demaria (2018) for review). Cell cycle arrest is a major characteristic of senescent cells and is regulated by induction of p53 (a tumor suppressor) and p21^Waf1/Cip1^ and p16^Ink4a^ cyclin‐dependent kinase inhibitors. Senescent cells not only undergo stable cell cycle arrest, but also exhibit morphological changes, including a flat cellular morphology, and upregulation of senescence‐associated β‐galactosidase (SA‐β‐gal) activity (Dimri et al. 1995). In particular, senescent cells display the senescence‐associated secretory phenotype (SASP), which is various secretory proteins such as chemokines, pro‐inflammatory cytokines, growth factors, and proteases (Watanabe et al. 2017). The SASP dynamically changes over time, has distinct features depending on the senescence state, and can elicit beneficial or deleterious effects according to the context.

Although senescent cells contribute to embryonic development and wound healing (Watanabe et al. 2017), they were recently recognized to be the major cause of aging and age‐related diseases (Baker et al. 2011), and therefore various approaches to remove these cells have been developed. For example, targeted elimination of senescent cells using senolytic small molecule drugs has been (Yosef et al. 2016; Zhu et al. 2015). Senostatics or senomorphics that can modulate the SASP regulatory network or inhibit deleterious components of the SASP have been developed (for a review of senolytic and senostatic drugs, see Di Micco et al. (2021)). The SASP plays an important role in immune surveillance, and immune cell‐mediated clearance of senescent cells has been proposed (Burton and Stolzing 2018). The SASP is a potent attractant to recruit immune cells, such as macrophages, neutrophils, T lymphocytes, and natural killer cells, which eliminate senescent cells. Therefore, the facilitation of immune cell‐dependent elimination of senescent cells via precise control of the SASP can be utilized to treat various diseases caused by senescent cells. However, the proposed approaches are mostly based on chemical drugs or biochemical methods and no other strategy has been developed.

Ultrasound is a promising non‐invasive tool for diagnosis and therapy. In therapeutic applications, ultrasound can be classified as either high‐ or low‐intensity. Various studies have reported the beneficial effects of low‐intensity pulsed ultrasound (LIPUS) on many types of tissue (Jiang et al. 2019). Depending on its experimental parameters, LIPUS stimulation induces angiogenesis in models of chronic myocardial ischemia and cardiac differentiation by stimulating cardiac mesoangioblasts (Hanawa et al. 2014). LIPUS stimulation also promotes DNA synthesis and cell proliferation in skin fibroblasts (Zhou et al. 2004). In addition, several studies demonstrated that LIPUS stimulation facilitates the treatment of cancer via sonodynamic therapy and ultrasound‐mediated gene delivery in combination with chemical compounds (Wood and Sehgal 2015).

Among these previously reported applications of LIPUS stimulation, we are particularly interested in studies demonstrating its role in regulating the secretion of inflammation‐associated cytokines (Li et al. 2003) and its promotion of wound healing and bone repair (Schortinghuis et al. 2005). Based on these reports, we hypothesized that LIPUS can modulate the secretion of SASP in senescent cells and thereby manipulate these cells or even immune cell attraction. In addition, LIPUS stimulation is advantageous for in vivo applications because it is already used in clinics and is easily applied in combination with other chemical and biochemical treatments.

The present study reports a regulatory mechanism underlying the clearance of senescent cells upon LIPUS stimulation. We demonstrate that LIPUS selectively stimulates senescent cells, but does not affect the cell cycle or cell proliferation. Furthermore, LIPUS stimulation specifically promotes the secretion of immune cell attraction markers such as CCL2, CXCL1, granulocyte macrophage colony‐stimulating factor (GM‐CSF), and granulocyte colony‐stimulating factor (G‐CSF), resulting in enhanced migration of monocytes/macrophages and upregulation of phagocytosis. Using an in vivo animal model of UV‐induced skin aging, we confirmed the effective removal of senescent cells through enhanced infiltrated macrophages via LIPUS treatment. Therefore, our results suggest that LIPUS can be a strategy to treat aging‐related diseases via immune cell‐dependent elimination of senescent cells.

## 2 Results

### 2.1 LIPUS Stimulation Does Not Induce Cell Cycle Arrest

**FIGURE 1:**
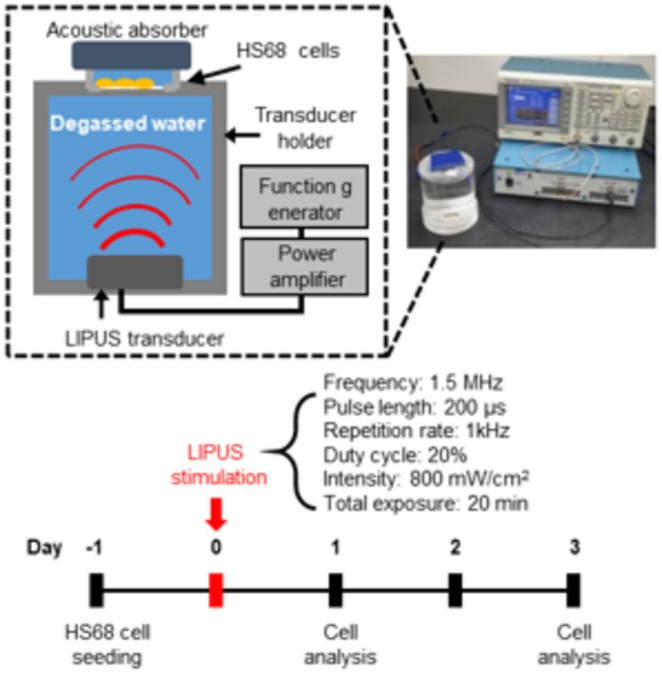
Experimental design for LIPUS stimulation. Schematic diagram of HS68 cell stimulation and experimental conditions for LIPUS stimulation. Cells were collected and analyzed on days 1 and 3 after LIPUS stimulation.

As a cellular senescence model, we induced replicative senescence in human HS68 cells by repeated cell culture. Population doubling time (PD) was calculated from the proliferation curve determined by the trypan blue exclusion assay (Figure S1A). We defined “late cells” (PD > 52 or passage 38–43) and “early cells” (PD < 28 or passage 16–20) and confirmed cellular senescence phenotypes, such as p16 and p21 expression, by immunofluorescence (Figure S1B). For LIPUS stimulation, we used a pulse length of 200 μs, a pulse repetition frequency of 1 kHz, and a total exposure time of 20 min (Figure 1). To determine the optimal condition for LIPUS stimulation, we varied the spatial‐peak temporal‐average intensity (*I*_spta_, see Figure S2 for calibration of *I*_spta_) from 30 to 1600 mW/cm^2^ and assessed the cellular senescence phenotype by SA‐β‐gal staining. The degree of SA‐β‐gal staining in late cells positively correlated with *I*_spta_ until 800 mW/cm^2^, whereas early cells barely showed SA‐β‐gal staining. Both early and late cells remained viable at an intensity of 1600 mW/cm^2^ (Figure S3). SA‐β‐gal activity is likely optimal under specific conditions. The effects of LIPUS stimulation can be vary depending on the type and physical properties of the cells and these effects can be maximized when the LIPUS condition matched with the cellular properties (Kureel et al. 2024). These data suggest that LIPUS stimulation selectively increases SA‐β‐gal in late cells.

**FIGURE 2:**
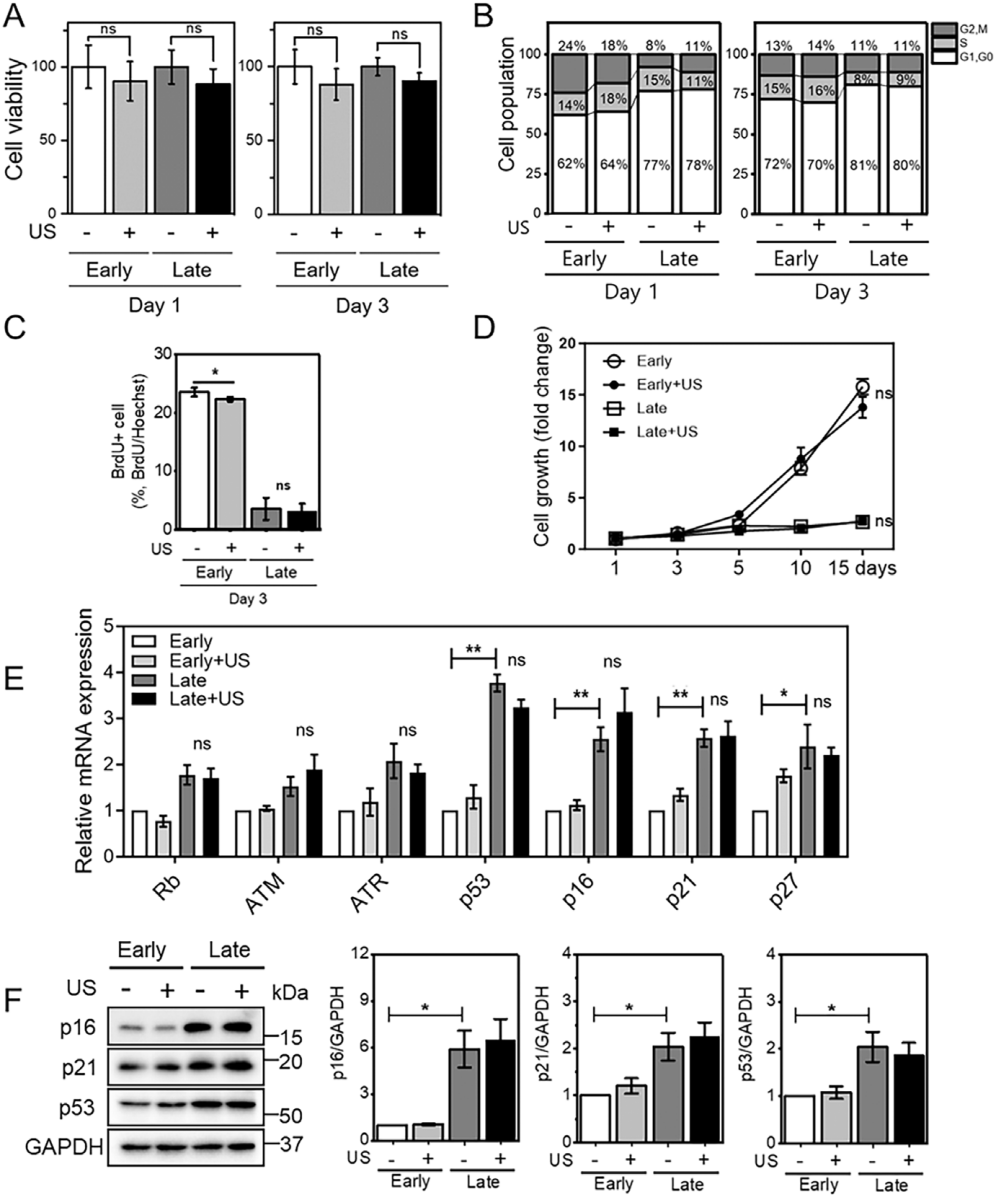
LIPUS stimulation does not affect the cell cycle. (A) Viability of HS68 cells stimulated with or without LIPUS on days 1 and 3. (B) Cell cycle analysis of HS68 cells stimulated with or without LIPUS on days 1 and 3. (C) BrdU assay of HS68 cells stimulated with or without LIPUS on day 3. (D) The cell growth rate of HS68 cells stimulated with or without LIPUS. (E) Real‐time qPCR and (F) Western blot analysis of cell cycle regulators. mRNA and protein expression of cell cycle regulators was assessed in early and late cells stimulated with or without LIPUS on day 3. Data represent means ± SEM of three independent experiments. *p < 0.05 and **p < 0.01.

Under this condition, we further examined other cellular senescence phenotypes. Cell viability was not significantly changed at 1 and 3 days after LIPUS stimulation (Figure 2A). Cell cycle analysis was performed by measuring DNA mass via fluorescence‐activated cell sorting (FACS) after propidium iodide (PI) staining. In general, replicative senescent cells irreversibly arrest in G1 phase of the cell cycle (Hernandez‐Segura, Nehme, and Demaria 2018). Consequently, the percentage of G1 phase in late cells was ~12% higher than in early cells, in the absence of LIPUS stimulation (Figure 2B). However, LIPUS stimulation did not affect any population of early or late cells. To further access proliferation, we also performed BrdU assay and measured growth rate (Figure 2C,D). LIPUS stimulation did not affect the proliferation rate of early or late cells.

To confirm that LIPUS stimulation did not alter the cell cycle, both mRNA and protein expression levels of various proteins involved in cell cycle regulation and DNA damage responses were measured. Although late cells showed enhanced expression of cell cycle regulation markers compared with the early cells, LIPUS stimulation did not significantly affect the expression of these markers (Figure 2E,F). Together, these results suggest that the LIPUS exposure conditions used in this study do not affect the cell cycle.

### 2.2 LIPUS Stimulation Enhances Senescence‐Associated Phenotypes (SA‐β‐Gal and SASP) and Migration/Phagocytic Activity of Macrophages

**FIGURE 3:**
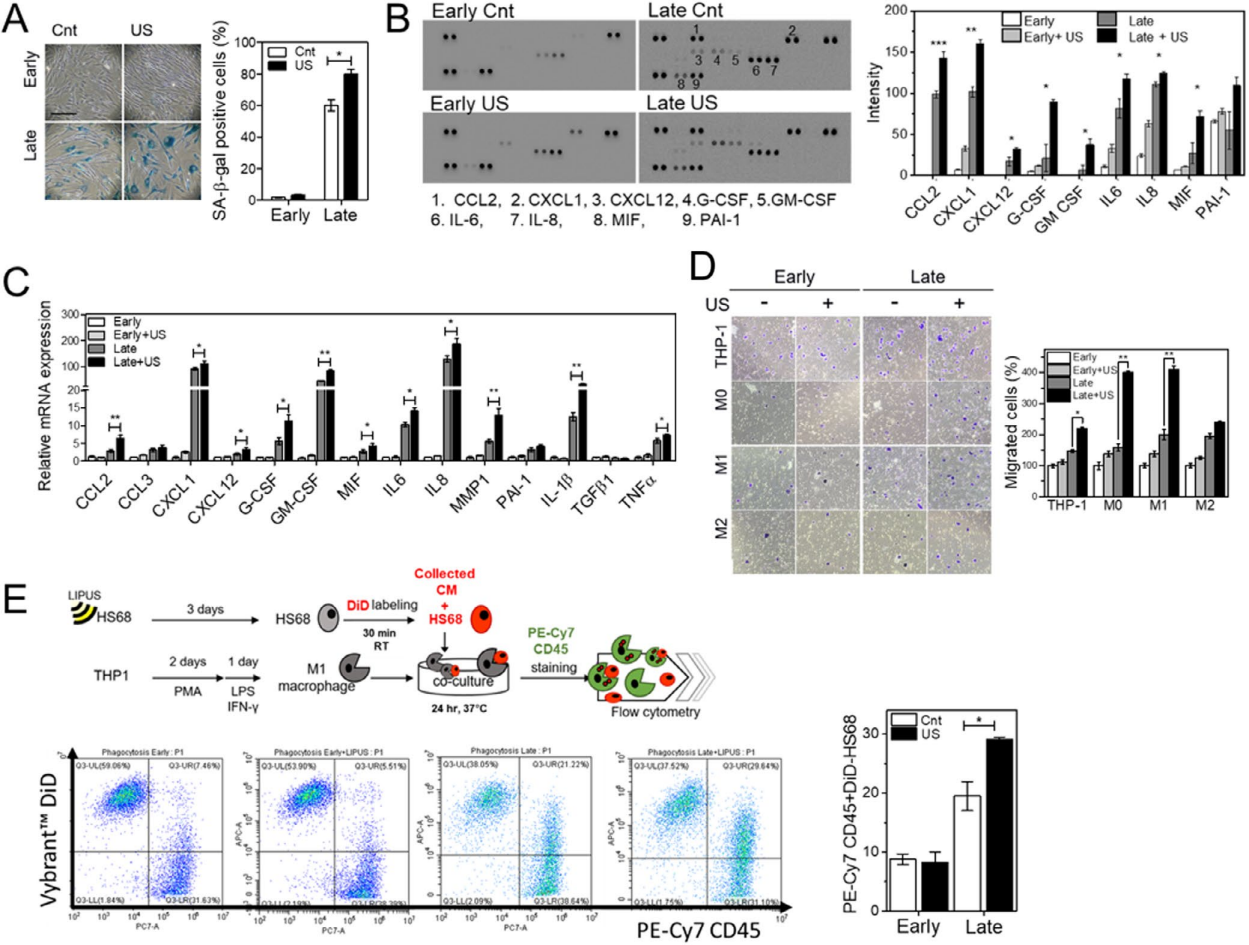
LIPUS stimulation enhances senescence‐associated phenotypes (SA‐β‐gal and SASP) and migration/phagocytic activity of macrophages. (A) SA‐β‐gal activity in HS68 cells stimulated with or without LIPUS on day 3. (B) Cytokine array analysis of early and late HS68 cells. The culture medium of HS68 cells stimulated with or without LIPUS was collected on day 3 (*N* = 3). (C) Real‐time qPCR analysis of SASP factors. Early and late HS68 cells stimulated with or without LIPUS were collected on day 3. mRNA expression of various cytokines involved in inflammation and immune cell recruitment was analyzed. (D) Transwell migration assay of monocytes, unpolarized macrophages, and M1 and M2 polarized macrophages. The culture medium of HS68 cells stimulated with or without LIPUS was collected on day 3 and added to the bottom chamber of the transwell. The migration of monocytes and macrophages from the upper chamber was measured by counting crystal violet‐labeled cells. (E) FACS analysis of phagocytosis. After LIPUS stimulation, DiD labeled‐HS68 cells were co‐cultured with M1 polarized macrophages in the presence of conditioned media for 24 h. Then, co‐cultured cells were collected and stained with FITC‐CD45 for M1 macrophages. Phagocytic activity was analyzed in 1 × 10^4^ DiD (DiD positive) cells via flow cytometry (*N* = 4). Experimental conditions for flow cytometry are shown in Figure S5. Data represent means ± SEM of independent experiments. *p < 0.05, **p < 0.01, and ***p < 0.001.

Although LIPUS stimulation barely affected the cell cycle in late cells, we next examined whether it changes senescence‐associated phenotypes, including SA‐β‐gal activity and expression of various cytokines, known as the SASP. As described above, LIPUS stimulation selectively promoted SA‐β‐gal activity in late cells, but early cells were not stained with β‐gal substrate (Figure 3A). Next, we explored whether LIPUS stimulation regulates the expression of SASP factors including inflammatory cytokines, chemokines, and growth factors. Specifically, we examined the expression of SASP factors in the following categories: immune cell attraction markers (CCL2, CCL3, CXCL1, CXCL12, G‐CSF, GM‐CSF), common markers of the SASP (IL‐6, IL‐8, MMP1, and PAI‐1) and inflammation markers (IL‐1β, TGF‐β, and TNF‐α). A cytokine array at day 3 demonstrated that LIPUS stimulation of late cells at day 3 selectively increased secretion of CCL2, CXCL1, CXCL12, G‐CSF and GM‐CSF, which act as attractant and activator for immune cells (Figure 3B). Also, common markers of SASP (IL‐6, IL‐8, MMP1, and PAI‐1) increased. Consistent with the cytokine array measurement, real‐time qPCR analysis revealed that LIPUS stimulation significantly increased the expression of these SASP factors at day 3 (Figure 3C).

The role of the SASP in senescent cells has been extensively studied (Watanabe et al. 2017). The SASP modulates the immune response by recruiting immune cells including natural killer cells, neutrophils, and macrophages, which are actively involved in the removal of senescent cells (Burton and Stolzing 2018; Prata et al. 2019). LIPUS stimulation specifically increased the expression of immune cell attraction markers in late cells; therefore, we further investigated its effects on immune cell responses using the transwell migration assay. THP‐1 monocytes were differentiated into M0 macrophages, and then macrophage polarization toward M1 and M2 was achieved by treatment with appropriate cytokines (see Materials and Methods and Figure S4A). The degree of polarization was validated by assessing specific marker expression using FACS and real‐time qPCR (Figure S4B,C). The culture medium of LIPUS‐stimulated late cells promoted migration of THP‐1 monocytes, M0 macrophages, and M1 macrophages, but not significantly of M2 macrophages (Figure 3D). Since SASP factors responsible for the activation of macrophages were increased, we further evaluated the phagocytic activity of M1 macrophages in the culture medium of LIPUS‐stimulated late cells. At 3 days after LIPUS stimulation, treated cells were labeled with a membrane staining dye, Vybrant DiD (DiD), and both labeled cells and the culture media were collected and seeded onto M1 macrophages and incubated for 24 h (Figure 3E). FACS analysis illustrated that the level of DiD‐positive M1 macrophages increased upon incubation with the culture medium of LIPUS‐stimulated late cells (Figure 3E and Figure S5). Taken together, these results suggest that LIPUS stimulation of late cells facilitates the recruitment of macrophages via increased expression and secretion of SASP factors, resulting in phagocytosis of late cells.

### 2.3 LIPUS Stimulation Enhances the SASP via the Reactive Oxygen Species‐Dependent p38‐NF‐κB Pathway

LIPUS stimulation selectively increased the migration of immune cells by upregulating SASP factor expression in late cells. Therefore, we investigated the mechanisms by which LIPUS stimulation increases SASP factor expression in late cells. One potential explanation for this is that senescent cells could induce paracrine senescence through the SASP or NOTCH signaling in surrounding cells. To test the possible paracrine effect, we measured SA β‐gal activity of both early and late cells after treatment with conditioned media (CM) collected on day 3 from the LIPUS stimulated cells (Figure S6A). Consistent with the previous measurements, late cells showed enhanced SA β‐gal activity, but the treatment of CM barely affected SA β‐gal activity (Figure S6B). Interestingly, early cells treated with CM from LIPUS‐treated late cells showed increased proliferation (Figure S6C). Therefore, it is likely that LIPUS stimulation does not induce paracrine senescence.

**FIGURE 4:**
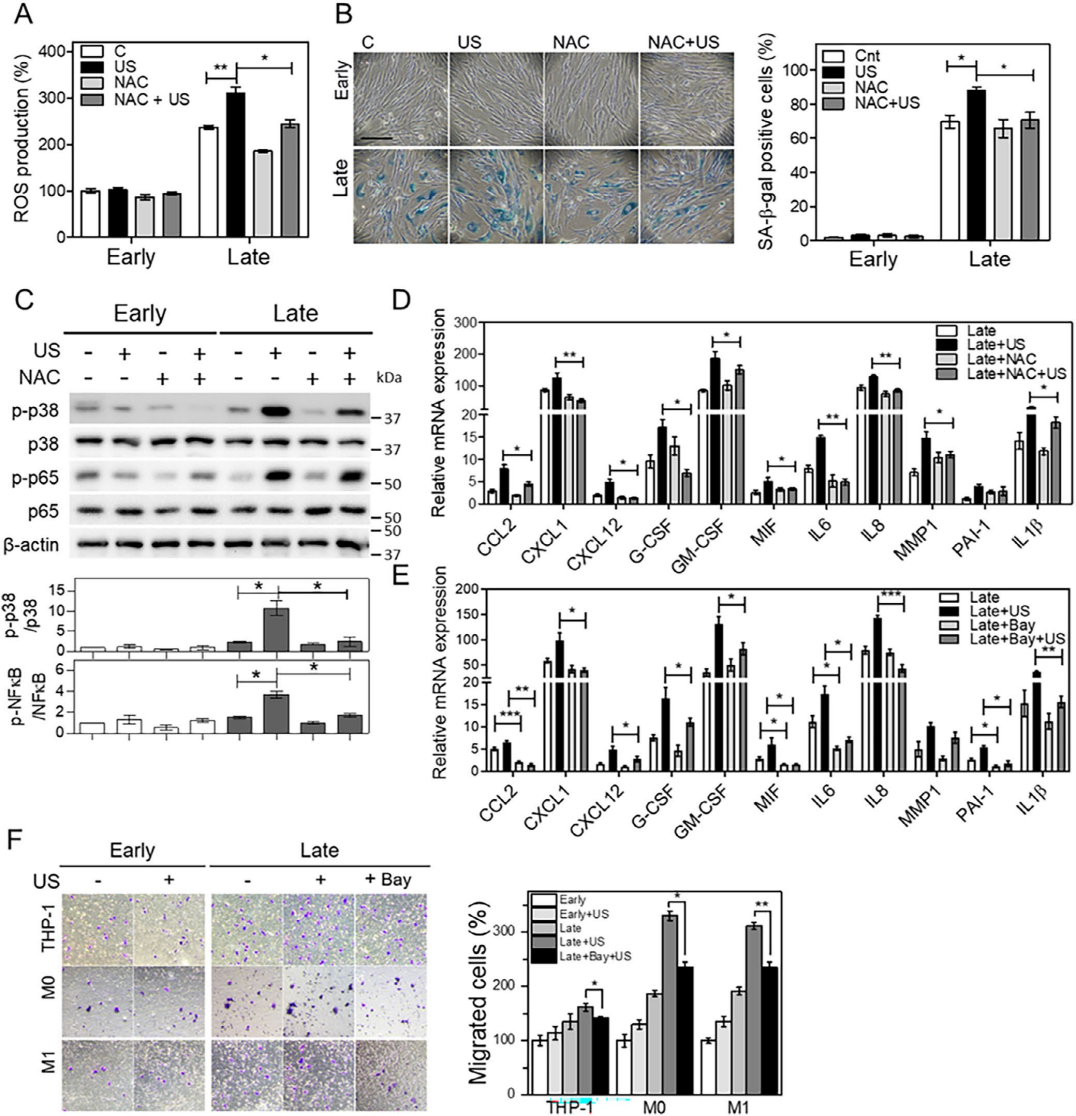
LIPUS stimulation enhances the SASP via the ROS‐dependent p38‐NF‐κB pathway. (A) ROS in HS68 cells stimulated with or without LIPUS. (B) SA‐β‐gal activity in HS68 cells treated with or without NAC treatment (5 mM) and stimulated with or without LIPUS on day 3. (C) Western blot analysis of p‐p38 and p‐NF‐κB with or without NAC treatment. The protein levels of p‐p38 and p‐NF‐κB were measured in early and late cells stimulated with or without LIPUS and normalized to the corresponding total protein levels (*N* = 3). (D) Real‐time qPCR analysis of IL‐6, IL‐1β, and GM‐CSF. Early and late HS68 cells treated with or without Bay 11‐7082 (NF‐κB inhibitor, 5 μM) and stimulated with or without LIPUS were collected on day 3. mRNA expression of various SASP factors was analyzed. (E) Transwell migration assay of monocytes, unpolarized macrophages, and M1 polarized macrophages. The culture medium of HS68 cells treated with or without Bay 11‐7082 and stimulated with or without LIPUS was collected on day 3 and added to the bottom chamber of the transwell. (E) Transwell migration assay of monocytes, unpolarized macrophages, and M1 polarized macrophages. The culture medium of HS68 cells treated with or without Bay 11‐7082 and stimulated with or without LIPUS was collected on day 3 and added to the bottom chamber of the transwell. The Migration of monocytes and macrophages from the upper chamber was measured by counting crystal violet‐labeled cells. Data represent means ± SD of three samples. *p < 0.05, **p < 0.01, and ***p < 0.001.

Previous studies have demonstrated that LIPUS stimulation enhances intracellular reactive oxygen species (ROS) production (Duco et al. 2016; Kaur et al. 2017). In addition, SASP factors reinforce senescence in an autocrine/paracrine manner by activating ROS production and downstream signaling (Nelson et al. 2018). Therefore, the effect of LIPUS on ROS generation was determined by flow cytometric analysis with the ROS‐sensitive fluorophore dihydroethidium (DHE). LIPUS stimulation of late cells significantly increased intracellular ROS generation compared with control late cells (Figure 4A). The ROS scavenger N‐acetyl‐l‐cysteine (NAC) was used to confirm that LIPUS increases SA‐β‐gal activity via ROS production. Consistent with the findings presented in Figure 3A, LIPUS stimulation increased SA‐β‐gal activity in late cells; however, this effect was significantly attenuated by pretreatment with NAC (Figure 4B).

NF‐κB, a key transcription factor that plays a pivotal role in SASP factor expression, directly regulates the induction of SASP factors (Di Micco et al. 2021). In addition, p38 activates SASP factor expression by increasing NF‐κB transcriptional activity and sustaining the SASP response (Freund, Patil, and Campisi 2011). Consequently, we examined whether LIPUS stimulation activates the p38‐dependent NF‐κB signaling pathway. Consistent with the previous report (Freund, Patil, and Campisi 2011). the phosphorylation levels of p38 and NF‐κB were higher in late cells than in early cells. LIPUS stimulation further enhanced the phosphorylation levels of p38 and NF‐κB in late cells, but NAC treatment attenuated both phosphorylation of p38 and NF‐κB, as well as SASP expression (Figure 4C,D) To confirm that LIPUS stimulation induces SASP factor expression via activation of NF‐κB, late cells were pretreated with Bay 11‐7082, an NF‐κB inhibitor. Real‐time qPCR revealed that pretreatment with Bay 11‐7082 significantly inhibited the upregulation of SASP factors in LIPUS‐stimulated late cells (Figure 4E). Finally, we investigated whether LIPUS stimulation of late cells enhances immune cell migration via NF‐κB activation. Migration of immune cells was increased by LIPUS stimulation of late cells, but this effect was significantly attenuated by pretreatment with Bay 11‐7082 (Figure 4F). These results suggest that LIPUS stimulation promotes SASP‐mediated immune cell migration by upregulating SASP factor expression via the ROS‐dependent p38‐NF‐κB pathway.

### 2.4 LIPUS Stimulation Induces Intracellular ROS Generation by Enhancing Transient Small Pore Formation (Sonoporation) and NADPH Oxidase (NOX)

**FIGURE 5:**
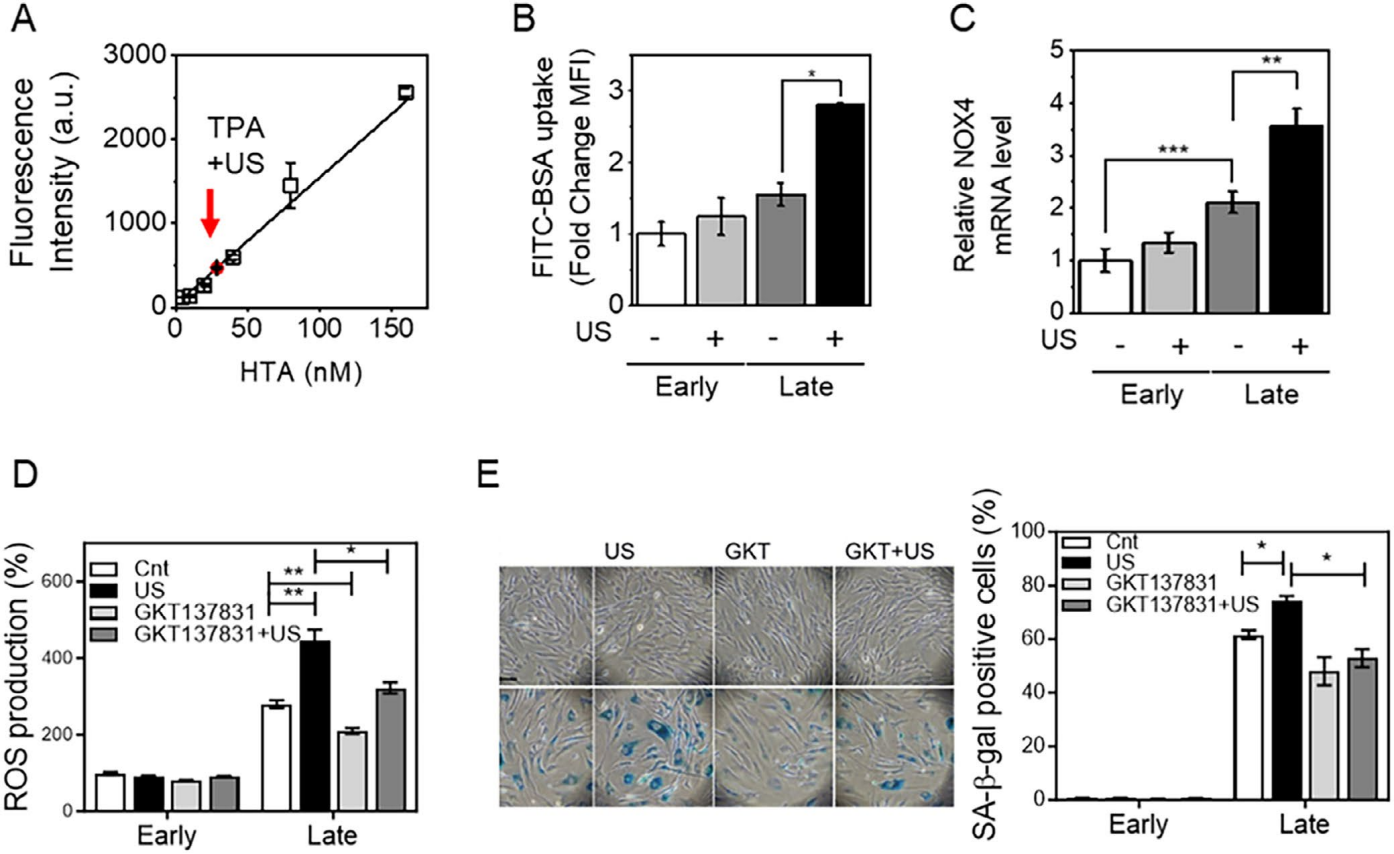
LIPUS stimulation induces intracellular ROS generation by enhancing membrane permeability (sonoporation) and NOX expression. (A) Measurement of hydroxyl radical generation upon LIPUS stimulation. HS68 cells were treated with PBS containing TPA (2 mM) and stimulated with LIPUS. Fluorescence of HTA formed by the reaction of TPA with hydroxyl radicals was measured using a fluorescence microplate reader. (B) Measurement of membrane permeability (sonoporation) based on uptake of FITC‐BSA. HS68 cells were incubated with FITC‐BSA (5 μM) and stimulated with LIPUS. The fluorescence of FITC‐BSA in HS68 cells was analyzed by FACS. (C) Real‐time qPCR analysis of NOX4. mRNA expression of NOX4 was measured in early and late cells stimulated with or without LIPUS. (D, E) To evaluate the role of NOX4 in ROS generation by LIPUS stimulation, cells were pre‐treated with a NOX4 inhibitor, GKT137831 (10 μM), prior to LIPUS stimulation. (D) ROS in HS68 cells in the presence or absence of GKT137831. Cells were stimulated with or without LIPUS and ROS was measured on day 3. (E) SA‐β‐gal activity in HS68 cells in the presence or absence of GKT137831. Cells were stimulated with or without LIPUS and activity was measured on day 3. Data represent means ± SEM of three independent experiments. *p < 0.05, **p < 0.01, and ***p < 0.001.

We explored how LIPUS stimulation selectively affects late cells via the ROS‐dependent p38‐NF‐κB pathway. We hypothesized that LIPUS stimulation (1) directly induces extracellular ROS generation, (2) promotes membrane oxidation and permeability, or (3) enhances intracellular ROS generation via NOX expression. Hydroxyl radicals (OH) and hydrogen peroxide (H_2_O_2_) can be directly formed by water sonolysis (Villeneuve et al. 2009) and can be detected by fluorescent probes such as 2′,7′‐dichlorodihydrofluorescein diacetate, DHE, and terephthalic acid (Montesinos et al. 2015). We used TPA to examine the degree of hydroxyl radical generation by measuring the fluorescence of hydroxyterephthalic acid (HTA), a reaction product of TPA with hydroxyl radicals, in media. Fluorescence was not significantly detected in the absence of LIPUS stimulation. However, HTA fluorescence was detected upon LIPUS stimulation, although the concentration of hydroxyl radicals, as determined by direct comparison with known concentrations of HTA, was not significant (< 50 nM) (Figure 5A).

Next, we determined the degree of transient small pore formation by measuring the uptake of the fluorophore‐labeled protein FITC‐BSA and plasmid DNA for mCherry expression from media by cells. ROS in media can directly modify or oxidize proteins and lipids in membranes, leading to perturbation of the plasma membrane structure and increased membrane permeability (Wang et al. 2017). Therefore, acoustic cavitation induced by LIPUS treatment can physically perturb the plasma membrane of late cells because the membrane composition and properties of these cells differ from those of early cells. Furthermore, it is well described that ultrasound can facilitate drug delivery or gene transfection by introducing transient small pores in the cell membrane (see Przystupski and Ussowicz (2022) for review). Various ultrasound parameters have been successfully used for the delivery of drug, DNA, and siRNA in vitro.

The population of fluorescently labeled cells increased when FITC‐BSA‐treated late cells were stimulated with LIPUS (Figure 5B), but uptake of FITC‐BSA by non‐stimulated late cells was negligible. Also, the uptake of FITC‐BSA by early cells was not significant, regardless of LIPUS stimulation. We further measured the degree of sonoporation using a mCherry plasmid DNA (pCMV‐mCherry) for mCherry expression. mCherry‐positive cells were observed in the case with LIPUS‐stimulated late cells, with a transfection efficiency of approximately less than 5% (Figure S7).

The level of intracellular ROS increased upon LIPUS stimulation (Figure 4A); therefore, we finally examined NOX expression. ROS‐generating NOX enzymes reside in specific lipid compartments called lipid rafts on the plasma membrane, and extracellular ROS can directly affect the formation and localization of lipid rafts, which is followed by NOX activation (Nordzieke and Medrano‐Fernandez 2018). Furthermore, physical perturbation of the membrane structure by LIPUS stimulation can influence the distribution and size of lipid compartments. NOX expression reportedly increases upon LIPUS stimulation (Kaur et al. 2017). Among various NOX isoforms (NOX1–5), mRNA expression of NOX4 was increased in late cells and further amplified by LIPUS stimulation, suggesting that NOX4 is involved in intracellular ROS production (Figure 5C). When cells were treated with NOX4 inhibitor GKT137831, intracellular ROS production stimulated by LIPUS diminished (Figure 5D). Furthermore, increased SA‐β‐gal activity in late cells upon LIPUS stimulation significantly attenuated by pretreatment with GKT137831 (Figure 5E). Lastly, we validated our observation with another in vitro model of senescence by cellular stress with H_2_O_2_ (Figure S8). Enhanced SA β‐gal activity following H_2_O_2_ treatment was further upregulated after LIPUS stimulation, but it was attenuated by NAC treatment (Figure S8A). Cell cycle analysis showed that cells became senescent upon H_2_O_2_ treatment, but LIPUS stimulation did not affect the cell proliferation rate (Figure S8B). LIPUS stimulation of H_2_O_2_ treated cells significantly increased intracellular ROS generation compared with that in control late cells (Figure S8C). The p38‐dependent NF‐κB signaling pathway was also demonstrated (Figure S8D). Real‐time qPCR analysis revealed that LIPUS stimulation significantly increased the expression of several SASPs in H_2_O_2_ treated cells at day 3 (Figure S8E). We also confirmed that the ROS enhanced by LIPUS stimulation was diminished by NAC treatment. Collectively, our data illustrate that LIPUS stimulation did not significantly induce extracellular ROS, but perturbed the membrane structure and organization, leading to enhanced membrane permeability and increased intracellular ROS generation via upregulation of NOX4 in late cells.

### 2.5 LIPUS Stimulation Contributes to Reducing Accumulated Senescent Cells in a UVA–Induced Skin Aging Model

**FIGURE 6:**
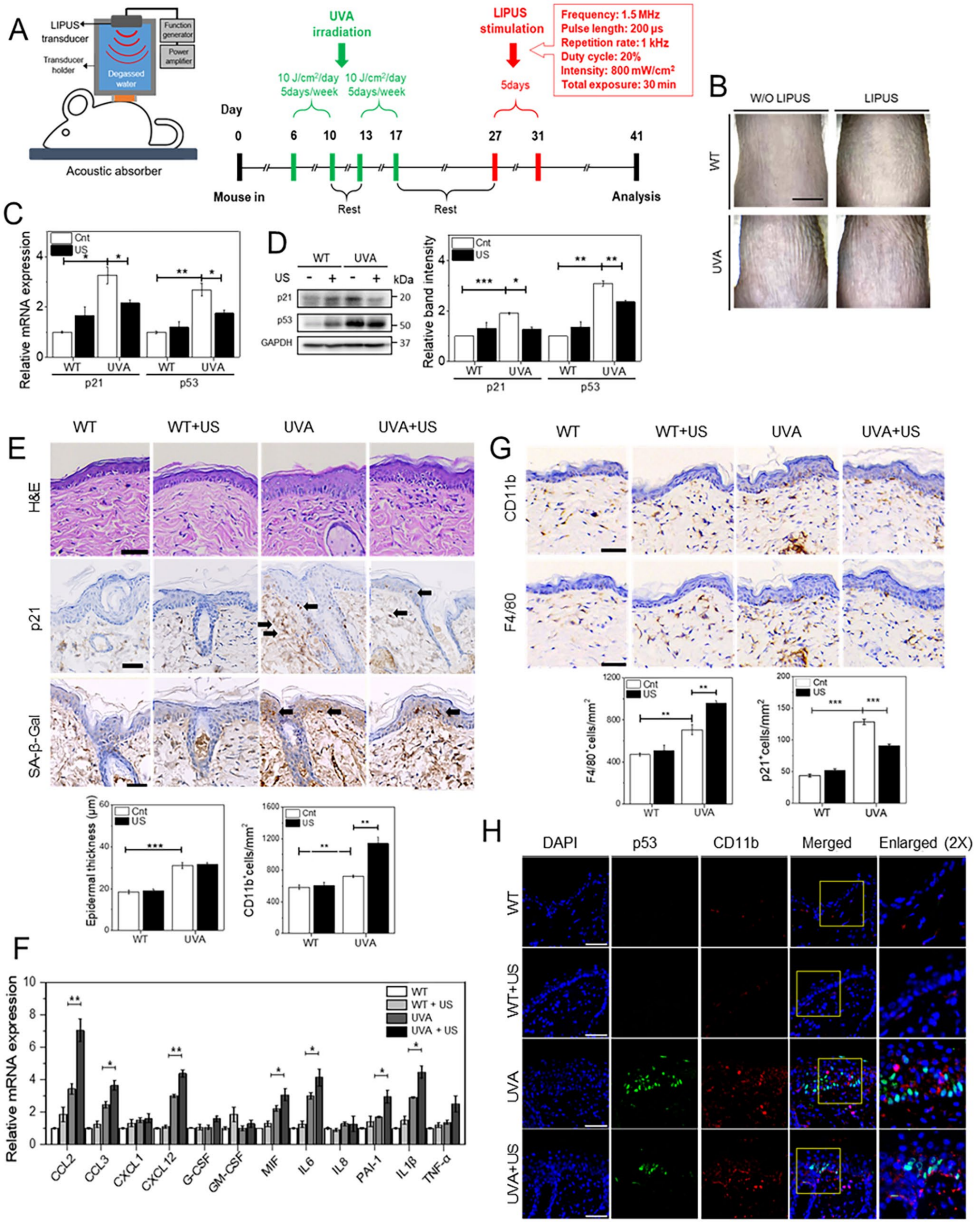
LIPUS stimulation contributes to reducing accumulated senescent cells in a UV‐ induced skin aging model. (A) Schematic diagram of LIPUS stimulation for mouse models and experimental conditions for UVA irradiation followed by LIPUS stimulation. (B) Photograph of mouse dorsal skin after UVA and LIPUS treatments. Both untreated and UVA‐ irradiated mice were either stimulated with or without LIPUS. Scale bar, 1 cm. (C) Real‐time qPCR and (D) western blot analysis of p21 and p53 from treated mouse dorsal skin. Both untreated and UVA‐ irradiated mice were either stimulated with or without LIPUS and treated mouse skin was collected for qPCR and Western blot as described in the Materials and Methods. (E) Representative images of hematoxylin/eosin (H/E), p21 and SA‐β‐gal immunohistochemistry (IHC) staining from treated mouse skin. Both untreated and UVA irradiated mice were either stimulated with or without LIPUS. The epidermal thickness was measured, and the number of p21 positive cells in a given area (mm^2^) were counted for quantification. Five different samples were used. Scale bar, 50 μm for H/E images and 20 μm for IHC images. (F) Real‐time qPCR analysis of SASP factors. Both untreated and UVA‐ irradiated mice were either stimulated with or without LIPUS. mRNA expression of various cytokines involved in inflammation and immune cell recruitment was analyzed. (G) Representative images of CD11b and F4/80 IHC staining from treated mouse dorsal skin. Both untreated and UVA‐ irradiated mice were either stimulated with or without LIPUS. The number of CD11b positive cells and F4/80 positive cells in a given area (mm^2^) were counted for quantification. Scale bar, 50 μm. (H) Representative immunofluorescence images of CD11b (red) and p53 (green) co‐staining from treated mouse dorsal skin. Both untreated and UVA‐ irradiated mice were either stimulated with or without LIPUS. Scale bar, 50 μm. Data represent means ± SEM of three independent experiments. *p < 0.05, **p < 0.01, and ***p < 0.001.

Finally, we investigated whether LIPUS stimulation can accommodate the reduction of senescent cells by regulating SASPs. As an in vivo model system, we used a UVA–induced skin aging mouse model considering the penetration efficiency of LIPUS through skin and tissue. Referred to the previous reports, the UVA irradiation protocol was optimized so that the p21 protein level in UVA‐irradiated mice was at least double that of control mice without UVA exposure (Kandan et al. 2020). We first used UVA‐induced senescent HS68 cells in vitro, demonstrating that UVA irradiation increased p16 and p21 levels and LIPUS stimulation promoted SA‐β‐gal activity and SASP factors (Figure S9). The dorsal region of the SKH1‐hr hairless mouse was irradiated with UVA (10 J/cm^2^) for 10 days, and then left for another 10 days (Figure 6A). LIPUS stimulation was applied on the same region for 5 days, and then the skin tissues were analyzed after 10 days of LIPUS stimulation (Figure 6A). We confirmed that HS68 cells were viable after 5 days of consecutive LIPUS stimulations in vitro (Figure S10A). Neither UVA nor LIPUS treatment influenced the weight of the body and major organs (Figure S10B,C). The clinical appearance of UVA–irradiated mice showed extensive and deep wrinkles on the dorsal region compared to control mice (Figure 6B). To confirm that UVA treatment induced skin aging, we measured both mRNA and protein expression levels of p21 and p53 from the skin tissue, and found that UVA irradiated mice showed enhanced p21 and p53 expression (Figure 6C,D). Hematoxylin/eosin (H/E) staining and Immunohistochemistry (IHC) studies showed that UVA treatment increased epidermal thickness of the skin tissue, and p21 and SA‐β‐gal positive cells were upregulated (Figure 6E and Figure S11). p21 and SA‐β‐gal positive cells were localized in both the epidermal and dermal region, consistent with the previous in vivo studies using UVA‐irradiated mice (Hung et al. 2014). Regardless of the cell type, it is likely that LIPUS stimulation under our experimental conditions promoted SASP in senescent cells.

Importantly, LIPUS treatment significantly reduced both mRNA and protein levels of p21 and p53 in a UVA‐induced skin aging model (Figure 6C,D), suggesting that LIPUS stimulation contributed to the decrease of senescent cells. To further explore whether the reduction of p21 and SA‐β‐gal positive cells after LIPUS treatment is caused by the clearance of senescent cells by immune cells, we examined the mRNA levels of SASP factors for immune cell attraction. Consistent with the in vitro measurements, markers for immune cell attraction, such as CCL2, CXCL1 and CXCL12 were further increased by LIPUS treatment (Figure 6F). Both CD11b positive monocytes and F4/80 positive macrophages were increased by UVA irradiation, and LIPUS stimulation further enhanced the infiltration of these cells (Figure 6G). Enhanced infiltration of CD11b‐positive cells into the region of p53‐positive senescent cells was observed by immunofluorescence staining, while the number of p53‐positive senescent cells decreased upon LIPUS treatment. Considering the in vivo microenvironment, where both senescent cells and residual immune cells can be stimulated together by LIPUS treatment, we also examined the effect of LIPUS stimulation on macrophage polarization. M1 specific cytokines, such as IL‐1β and CCR7, were upregulated when macrophages were polarized to M1 by LPS/INF‐γ, and further enhanced by LIPUS treatment (Figure S12). These data illustrated that LIPUS treatment can stimulate immune cell infiltration for the removal of senescent cells, as well as modulate cytokine production of M1. Together, these data suggest that senescent cells could be eliminated by macrophage infiltration via LIPUS stimulation.

## 3 Discussion

This study demonstrated that LIPUS selectively stimulates senescent cells without affecting the cell cycle and specifically promotes the secretion of cytokines involved in immune cell migration and activation, resulting in enhanced removal of senescent cells by phagocytosis. Quantification of extracellular ROS, membrane permeability by sonoporation, and intracellular ROS revealed that membrane perturbation, as evidenced by enhanced protein and DNA delivery, underlies intracellular ROS generation via NOX4 expression, leading to activation of the ROS‐dependent p38‐NF‐κB signaling pathway.

Exposure to weak‐pressure ultrasound alone has a very limited capacity to activate an immunological response (Scarponi et al. 2009). LIPUS can enhance blood vessel permeability, reduce tumor volume, and elevate the anticancer immunological response (Shibaguchi et al. 2011). However, the effects of LIPUS on immune system‐mediated clearance of senescent cells have not been studied. To the best of our knowledge, this is the first study to report that in the absence of any reagent, LIPUS selectively induces expression of immune cell attractant and activation cytokines in senescent cells without affecting the cell cycle, leading to the recruitment of immune cells and the eventual removal of the senescent cell by phagocytosis.

Despite the extensive applications of LIPUS mentioned above, its effect on senescent cells is barely studied. Recent work by Kureel et al. demonstrated that LIPUS can stimulate both normal cells and senescent cells (Kureel et al. 2024). In specific, they claimed that cytokines involved in cell growth and proliferation were released from normal cells. At the same time, rejuvenation of senescent cells could be achieved by calcium ion‐ dependent mTOR signaling pathway. It is noteworthy that the ultrasound conditions that Kureel et al. usedare quite different (frequency of 33 kHz, pulse length, of 1.5 s and repetition rate of 0.333 Hz) compared to most of other LIPUS studies (e.g. Ultrasound frequencies of 1–3 MHz, pulse repetition frequency of 1 kHz, and spatial average and temporal average intensity (*I*_spta_) of 30 mW/cm^2^ are widely used (Jiang et al. 2019)). It is important to note that LIPUS induces varying cellular effects depending on its specific parameters as well as the properties of the cells used. For example, LIPUS stimulation (1.5 MHz, 200 μs, repetition rate 1 kHz, *I*_spta_ 30 mW/cm^2^) increased the proliferation of human fibroblast via Rho/ROCK pathway (Zhou et al. 2004), although Kureel et al. found no such effects in non‐senescent, proliferating cells (Kureel et al. 2024). Rather, Kureel et al. demonstrated that LIPUS enhanced proliferation specifically in senescent cells through Rho kinase activity, indicating that cellular properties (non‐senescent vs. senescent) should be also considered.

The mechanisms underlying the effects of LIPUS stimulation are not fully understood, although several studies have attempted to determine its mechanism‐of‐action in different cells. To elucidate the detailed mechanism underlying activation of the ROS‐dependent p38‐NF‐κB pathway in senescent cells, we explored how LIPUS induced intracellular ROS generation under our experimental conditions. Senescent cells display metabolic changes in lipid biosynthesis and distinct alterations in lipid composition, all of which affect the chemical and physical properties of the plasma membrane (Millner and Atilla‐Gokcumen 2020). Furthermore, lipid rafts, which are sphingolipid‐ and cholesterol‐rich domains, and transmembrane proteins such as caveolin‐1, a key player in oxidative stress signaling, are altered in senescent cells, resulting in increased sensitivity to ROS (Nordzieke and Medrano‐Fernandez 2018; Zou et al. 2011). We previously reported alteration in membrane fluidity, hydrophobicity, and lipid composition (including ganglioside GM1 levels ceramide‐rich lipid rafts) in replicative senescent HS68 cells, which were also used in this study (Wi et al. 2021). In the previous study, we demonstrated that membrane fluidity decreases (or rigidity increases) and the membrane becomes more hydrophobic with an increased ceramide‐rich lipid raft as HS68 cells undergo senescent. On the other hand, a recent paper by Rong et al., showed that the rigidity of the extracellular matrix (ECM) plays an important role in sonoporation with microbubbles, revealing that cells on rigid substrates (with enhanced cell spreading due to more focal adhesions and F‐actin fibers) exhibit higher sonoporation efficiency with smaller pore formation (Rong et al. 2020). Given the enhanced cell spreading and increased membrane rigidity in senescent cells, it is reasonable to assume that senescent cells may be more susceptible to LIPUS stimulation.

Acoustic cavitation induced by LIPUS treatment can induce water sonolysis, leading to the production of hydroxyl radicals and hydrogen peroxide in the solution, although the experimental conditions differed between these previous studies and the present study (Villeneuve et al. 2009). The production of hydroxyl radicals is proportional to the acoustic power applied to the medium. However, the level of extracellular ROS generated upon LIPUS stimulation was not significant (< 50 nM in Figure 5A) under our experimental conditions in comparison with typical ROS treatment used to induce senescence (50–800 μM H_2_O_2_) (Chen, Ozanne, and Hales 2007). Consistently, LIPUS stimulation did not affect other senescence markers or the cell cycle (Figure 2C–E). Instead, the distinct membrane properties of senescent cells might be attributable to enhanced membrane permeability, which increases intracellular NOX expression (Figure 5B–E). Due to mechanical and oxidative stress caused by LIPUS stimulation, the intracellular ROS‐generating enzyme NOX is activated, leading to ROS production. NOX is a transmembrane enzyme in the plasma membrane, and therefore membrane perturbation by LIPUS also stimulates NOX enzymes (Lambeth and Neish 2014). In addition, LIPUS stimulation induces differentiation of pre‐osteoblasts via NOX2‐ and NOX4‐mediated ROS generation (Kaur et al. 2017). Similarly, we found that the elevation of intracellular ROS via enhanced NOX4 expression activated the p38‐NF‐κB pathway (Figure 5C–E).

Regarding the downstream signaling of intracellular ROS, the p38‐NF‐κB signaling pathway, which is independent of the persistent DNA damage response was examined. Phosphorylation of p38 and NF‐κB was significantly increased in LIPUS‐stimulated late cells (Figure 4C). In addition, LIPUS stimulation increased the level of intracellular ROS (Figure 4A), which act as signaling molecules to activate downstream signaling pathways including the MAPK and NF‐κB pathways (McCubrey, Lahair, and Franklin 2006). NAC, a ROS scavenger, impaired the LIPUS‐stimulated expression of NF‐κB target genes (Figure 4D,E). Therefore, our results suggest that LIPUS stimulation upregulates SASP factors in senescent cells via the ROS‐dependent p38‐NF‐κB pathway.

In general, senescent cells irreversibly undergo G1 cell cycle arrest due to upregulation of p16 and p21. We showed that late cells were not only arrested in G1 phase, but also displayed higher expression of p16 and p21 than early cells (Figure 2B,E). However, LIPUS selectively stimulated late cells and enhanced the SASP without affecting G1 arrest or p16/p21 expression. The regulatory mechanisms of SASP induction have not been fully elucidated. However, it has been suggested that the SASP is not induced by p16‐ or p21‐dependent cell cycle arrest (Coppe et al. 2008). In addition, p16 expression is sufficient to induce cell cycle arrest, but does not induce or modify the SASP (Coppe et al. 2011). Likewise, SA‐β‐gal‐positive cells can be negative for other canonical makers involved in senescence (Piechota et al. 2016). Furthermore, SA‐β‐gal staining results can be subjective, as this assay can be influenced by various experimental conditions, including reaction time, temperature, and pH. Therefore, it is likely that both the SASP and SA‐β‐gal do not always correlate directly with cell cycle markers and can be independently upregulated regardless of cell cycle arrest, as shown by our data.

Macrophages play important roles in wound healing, which involves inflammation, proliferation, and remodeling (Ferrante and Leibovich 2012). They also have the pivotal function of clearing senescent cells. Once senescent cells produce SASP factors, macrophages are recruited to clear these cells (Burton and Stolzing 2018). Unique features of the SASP with distinct functions appear to be displayed in different senescent states, cell types and senescence inducers (Watanabe et al. 2017). For example, IL‐6 and IL‐1 are expressed in senescent fibroblasts and directly affect neighboring cells that express receptors for these molecules (paracrine fashion). Chemokines such as those belonging to the CXCL family can activate a self‐amplifying secretory network and thereby reinforce growth arrest (autocrine fashion). Inflammatory cytokines including GM‐CSF and G‐CSF are involved in macrophage infiltration and activation (Prata et al. 2019). Interestingly, our study revealed that LIPUS stimulation promoted the expression of SASP factors (Figure 3B,C). The upregulation of SASP factors by LIPUS stimulation was consistently observed across different senescent models, including ROS‐induced and UVA‐induced senescence (Figures S8E and S9C), strongly supporting our conclusions. LIPUS stimulation significantly increased the secretion of cytokines associated with immune cell attraction, consequently enhancing the migration of M1 macrophages more than that of M2 macrophages. Furthermore, the culture medium of LIPUS‐stimulated senescent cells enhanced the phagocytic activity of M1 macrophages (Figure 3E).

We demonstrated that membrane perturbation upon LIPUS stimulation selectively stimulated senescent cells, probably due to the distinct molecular characteristics of their plasma membrane. Once SASP factors are generated and secreted, they can promote autocrine or paracrine signaling, thereby amplifying the secretion of SASP factors in neighboring cells, although we did not observe a paracrine effect of the conditioned media from the LIPUS‐stimulated cells. Furthermore, excessive levels of ROS can overwhelm the ROS‐scavenging ability of non‐regenerative cells (Saxena et al. 2019), but this situation did not arise in our experimental conditions because only a low level of ROS was detected (Figure 5A) after LIPUS stimulation. Instead, it is likely that ROS‐activated NF‐κB signaling, upon LIPUS stimulation, contributes to increased SASP factor secretion and senescence in bystander cells, as reported previously (Nelson et al. 2012).

To further validate our findings, we evaluated the effect of LIPUS stimulation using a UV‐induced skin aging model. The effects of LIPUS treatments are determined by various parameters such as frequency, pulse length, repetition rate, and intensity. In the case of in vitro experiments, ultrasound usually passes through degassed water to minimize the attenuation of ultrasound waves to the sample. However, the efficiency of LIPUS penetration through skin and tissue changes in vivo. Considering these limitations, we selected the UVA‐induced skin aging model as our in vivo system. Under our experimental conditions, SA‐β‐gal, p21, or p53 positive senescent cells accumulated after UVA treatment (Figure 6C,D). Since these accumulated senescent cells secreted SASPs (Figure 6F), the amount of CD11b or F4/80 positive macrophages increased, suggesting that the recruitment of immune cells by SASP was effective under our conditions after UVA treatment, and was further upregulated by LIPUS stimulation (Figure 6G,H). However, both mRNA and protein levels of p21 and p53 were decreased upon LIPUS stimulation, indicating a reduction in senescent cells (Figure 6C,D). Indeed, the upregulated SASP by LIPUS stimulation (Figure 6F) could have contributed to the increase in CD11b and F4/80 positive macrophages in the UVA‐treated region (Figure 6G), and their co‐localization with senescent cells stained with p53 increased (Figure 6H). At this point, it is not clear why the mRNA expression levels of SASPs remained high after the clearance of senescent cells with LIPUS treatment. It is possible that these cytokines originated from enhanced infiltrated macrophages and other cell types (such as keratinocytes, endothelial cells, and other immune cells) that were stimulated by LIPUS treatment. Meanwhile, since LIPUS stimulation can affect not only senescent cells but also immune cells or macrophages in the same region, we also evaluated the effect of LIPUS on macrophages by measuring the changes in cytokines related to their polarization in vitro. When LIPUS was applied to M1 macrophages, cytokine secretion such as IL‐1B, TNF‐alpha, and CCR7 increased (Figure S12). These results imply that LIPUS not only increases the SASP in senescent cells, but also changes the cytokine productions of M1 and M2 macrophages.

Accumulation of senescent cells can contribute to senescence‐related inflammation, which is pathogenic in many chronic diseases, such as fibrosis, osteoarthritis, osteoporosis, and Parkinson's disease (Di Micco et al. 2021). Furthermore, senolytic compounds are being developed to treat age‐associated dysfunctions. Therefore, targeting senescent cells is a promising potential approach to treat age and age‐related (Baker et al. 2011). In addition to the development of senolytic drugs, immunotherapy targeting senescent cells is a new promising strategy to treat age‐related diseases because the SASP of senescent cells induces an immune response (Burton and Stolzing 2018), and various approaches, including senolytic CAR‐T and vaccines have been developed (Amor et al. 2020; Hasegawa et al. 2023). On the other hand, ultrasound treatment has been extensively utilized in clinical applications, although most are focused on sonoporation‐dependent cancer therapy (Woodall et al. 2020). Our study suggests that LIPUS stimulation enhances the migration of immune cells and increases phagocytosis of senescent cells both in vitro and in vivo (Figures 3D,E and 6G,H). Because LIPUS stimulation can be easily adapted for clinical applications, it can be applied as a therapeutic strategy to remove senescent cells. Furthermore, treatment with other senolytics in combination with LIPUS stimulation can be a potential strategy to prevent or treat age‐associated diseases via clearance of senescent cells due to activation of the immune system. Still, several issues need to be considered for therapeutic application. First, LIPUS stimulation can be limited due to its penetration efficiency, and further optimization of LIPUS parameters may be necessary for deeply located tissues. Second, the immune system of aged individuals may not be fully functional, and it is possible that the enhanced infiltration of macrophages may not be effective in clearing senescent cells in older individuals.

In conclusion, we showed that LIPUS selectively stimulates senescent cells and promotes secretion of SASP factors, leading to enhanced immune cell migration. Consequently, LIPUS stimulation induces the elimination of senescent cells both in vitro and in vivo. Taken together, our findings reveal the effect of LIPUS on cellular senescence and our developed technique can be applied to treat senescence‐related diseases by enhancing the removal of senescent cells.

## 4 Experimental Procedures

Details of materials and methods are described in the supplementary information.

## Author Contributions

S. Y. Kim, H. Kim, and K. J. Pahk designed the research. H. Gwak, S. Hong, S. H. Lee, I. W. Kim, Y. Kim, K. J. Pahk, and S. Y. Kim performed research. H. Gwak, S. Hong, S. H. Lee, I. W. Kim, Y. Kim, S. Y. Kim and K. J. Pahk and H. Kim analyzed data. All of the authors wrote the manuscript together.

## Conflicts of Interest

The authors declare no conflicts of interest.

## Supporting information


Data S1.

## Data Availability

The data that support the findings of this study are available from the corresponding author upon reasonable request.
