# Supplementary material for: Low‐Intensity Pulsed Ultrasound Treatment Selectively Stimulates Senescent Cells to Promote SASP Factors for Immune Cell Recruitment
Source: Aging Cell. 2025 Jan 16;24(5):e14486. doi: 10.1111/acel.14486 (PMC12073891; doi:10.1111/acel.14486)
Supplement: Supplementary file 1 — Data S1. [file ACEL-24-e14486-s001.docx]

**SUPPLEMENTAL INFORMATION**

**Low-intensity pulsed ultrasound treatment selectively stimulates senescent cells to promote SASP factors for immune cell recruitment**

HyeRan Gwak^1^, Seoyoung Hong^1^, Su Hyun Lee^1^, In Woo Kim^1^, Yonghan Kim^1^, Hyungmin Kim^2, 3, 4 *^, Ki Joo Pahk^4, 5*^, and So Yeon Kim^1, 3, 4*^

^1^Chemical and Biological Integrative Research Center, Biomedical Research Division, Korea Institute of Science and Technology, Seoul, Republic of Korea

^2^Bionics Research Center, Biomedical Research Division, Korea Institute of Science and Technology, Seoul, Republic of Korea

^3^Division of Bio-Medical Science and Technology, KIST school, Korea University of Science and Technology (UST), Seoul, Republic of Korea

^4^KHU-KIST Department of Converging Science and Technology, Kyung Hee University, Seoul, Republic of Korea

^5^Department of Biomedical Engineering, Kyung Hee University, Yongin, Republic of Korea

**Correspondence should be addressed to S. Y. K. (soyeonkim@kist.re.kr), K. J. P. (kjpahk@khu.ac.kr) and H. K. (hk@kist.re.kr)**

**Email:** soyeonkim@kist.re.kr, kjpahk@khu.ac.kr, hk@kist.re.kr,

**ORCID ID:** So Yeon Kim (0000-0001-9167-5182)

**MATERIALS AND METHODS**

**Materials**

Poly-L-lysine, Crystal violet, terephthalic acid (TPA), 2-hydroxyterephthalic acid (HTA), N-acetyl-L-cysteine (NAC), Bay 11-7082, hydrogen peroxide solution (H_2_O_2_), and DNase I were purchased from Sigma-Aldrich (USA). FDA, Propidium iodide (PI), Hoechst33342 and MTT (3-(4,5-Dimethylthiazol-2-yl)-2,5-Diphenyltetrazolium Bromide) were purchased from Invitrogen (USA). FITC labeled BSA was obtained from Sigma-Aldrich (USA). GKT137831 was purchased from Selleckchem (USA). Vybrant DiD was purchased from Thermo Fisher Scientific (USA). Primary antibodies for p16 (sc-56330), phosphor-p38 (sc-166182) and p38 (sc-7972) were purchased from Santa Cruz Biotechnology (USA). phosphor-NF-kB (Ser536, 3033) and NF-kB (8242) were purchased from Cell signaling (USA). p21 primary antibody (ab109520) was purchased from Abcam (USA). BrdU, FITC-CD68 (333805), PE-CD80 (305217), and APC-CD206 (321109), and PE-Cy7 CD45 (304016) were purchased from Biolegend (USA). Anti-BrdU antibody was obtained from ABclonal (USA). For animal experiments, primary antibodies for p53 (ab241566), p21 (ab108099), and CD11b (ab133357) were obtained from Abcam (USA). Antibody for F4/60 (#70076) and SA-β-gal staining kit (#9860) was purchased from Cell Signaling (USA). Anti-mouse Alexa 488 and anti-rabbit Alexa 594 secondary antibody were obtained from Thermo Fisher Scientific (USA). β-actin (MA5-15739) was purchased from Sigma-Aldrich (USA). As for the cytokines for THP differentiation and macrophage polarization, phorbol 12-myristate 13-acetate (PMA) was obtained from Tocris Bioscience (UK). Lipopolysaccharide (LPS) was purchased from Sigma-Aldrich (USA). Interfereon-γ (INF-γ), interleukin-4 (IL-4) and interleukin-13 (IL13) were purchased from PeproTech (USA).

**Cell culture**

Human skin fibroblasts HS68 cells were obtained from the ATCC (USA). HS68 cells were maintained in DMEM (Welgene, Korea) with 10% fetal bovine serum (FBS, Gibco, USA) at 37°C under atmosphere of 5% CO_2_, respectively. Population doubling time (PD) of HS68 cells was calculated from the equation, $PD= \frac{duration \times log2}{\log\left( Final cell number \right)-\log(Initial cell number)}$and “late cell” (PD > 52, passage between 38 and 43) and “early cell” (PD <28, passage between 16 and 20) were used for all experiments. For oxidative stress induced senescence, HS68 cells were treated with 100 μM of H_2_O_2_ (Chen, Ozanne, & Hales, 2007). Briefly, 100 μM of H_2_O_2_ were treated for 2 h and then the medium was replaced. After 3 days (day 3), treated cells were re-plated on a confocal dish, incubated 24 h and then added H_2_O_2_ for a second time (day 4). In case for LIPUS treatment, cells were stimulated after 3 days (day 7) of second H_2_O_2_ treatment. Either LIPUS treated or non-treated cells were analyzed after 3 days (day 10).

Human monocyte THP-1 cells from the Korean Cell Line Bank were cultured in RPMI-1640 medium supplemented with 10% FBS at 37 °C under atmosphere 5% CO_2_. To generate M0 macrophages (M0), THP-1 cells were treated with 20 nM PMA for 48 h. M0 macrophages were polarized into M1 macrophages by incubation with 20 ng/ml of IFN-γ and 20 ng/ml of LPS for 24 h and M2 macrophage obtained by stimulation with 40 ng/ml of IL-4 and 20 ng/ml of IL-13 for 24 h.

**LIPUS stimulation**

HS68 cells (1x10^4^ cells /cm^2^) were seeded on a poly-L-lysine (0.1 mg/mL) coated 35 mm-sized confocal dish (SPL, Korea) and incubated for 24 h. The LIPUS experimental setup shown in Fig. 1 was used to investigate the effects of LIPUS on replicative senescence. A 1.5 MHz unfocused ultrasound transducer (diameter = 20 mm, #S19A001, DONG IL Technology Ltd, Korea) was placed on the bottom of a customized transducer holder which was filled with degassed water. A 35 mm-sized confocal dish containing HS68 cells was placed on the opposite end to the transducer. An acoustic absorber (AptFlex F28, Precision Acoustics Ltd, UK) was used in order to minimize ultrasonic reflections. The LIPUS source was driven by a function generator (33500B, Keysight, USA) via a power amplifier (210L, Electronics & Innovation, USA). The following LIPUS exposure conditions were employed in the present study: a pulse length of 200 μs, 1 kHz pulse repetition frequency and a total exposure time of 20 min with spatial-peak temporal-average intensities (I_spta_) of 30, 400, 800 and 1600 mW/cm^2^. These acoustic intensity values were experimentally measured at the center surface of the cell dish along the transducer’s axial axis using a calibrated hydrophone (HNR-500, ONDA, USA) and a digital oscilloscope (DPO 4104, Tektronix, USA) sampling at 0.1 GHz, as shown in Fig. S7.

**Cell viability assay**

HS68 cells (1x10^4^ cells /cm^2^) were seeded on a poly-L-lysine (0.1 mg/mL) coated 35 mm-sized confocal dish (SPL, Korea). Treated cells were washed with PBS and detached from the plate using Trypsin-EDTA (TE, Gibco, USA). Then, cells were resuspended in PBS and then mixed with Trypan blue Sigma, USA) in a 1:1 mixture. The mixed solution was put into the hemocytometer, and the number of viable cells was selectively counted.

**Cell growth rate measurements**

HS68 cells were seeded on a poly-L-lysine (0.1 mg/mL) coated 35 mm-sized confocal dish (SPL, Korea). 1x10^3^ of treated cells were plated onto a 96-well-plate. After indicated time, cells were incubated with 50 μL MTT (2 mg/mL) for 3 hours at 37 °C under atmosphere 5% CO_2_. Then, formazan was dissolved using 100 μL of DMSO and optical density at 540 nm was measured by a multi-scan spectrophotometer.

**SA β-Gal staining**

SA β-Gal Staining Kit (Cell signaling, USA) was utilized according to the instructions by manufacture. In brief, treated cells were washed with PBS and fixed with 4% paraformaldehyde (PFA, Biosesang, Korea) for 15 minutes. Then, cells were washed with PBS for three times and β-gal staining solution was added. Cells in culture dish were sealed and placed in the incubator at 37°C overnight. The degree of β-gal activity, blue color produced by β-gal was observed with a light microscope (Nikon, Japan). Cell nucleus was labeled with Hoechst 33342 (1 µg/mL in PBS) to count the number of cells. SA-β-Gal positive cells were quantified using the ‘senescence counter’ plug-in developed by Lozano-Gerona et al (Lozano-Gerona & Garcia-Otin, 2018).

**Cell cycle analysis by FACS**

HS68 cells (1x10^4^ cells /cm^2^) were seeded on a poly-L-lysine (0.1 mg/mL) coated 35 mm-sized confocal dish (SPL, Korea). Treated cells were washed with PBS and detached with TE (Gibco, USA). Cells were fixed with 70% ethanol solution and incubated for 12 h at 4°C. After washing with PBS, DNA was stained by adding RNase (50 μg/mL, ELPIS biotech, Korea) and PI (25 μg/mL, Thermo Fisher Scientific, USA). The amount of DNA stained by PI in each cell was measured by Flow cytometer (CytoFLEX, Beckman, USA) and analyzed using the FlowJo program (FlowJo, USA).

**BrdU assay**

HS68 cells (1x10^4^ cells /cm^2^) were seeded on a poly-L-lysine (0.1 mg/mL) coated 35 mm-sized confocal dish (SPL, Korea). Treated cells were incubated with 10 μM of BrdU for 24 hours at 37°C. After incubation, cells were fixed using 4% of PFA for 20 min and washed with PBS. Cells were permeabilized using 0.2 % Triton X-100, washed with PBS and incubated with 2% FBS in PBS for 15 min. Then, DNA was denatured by DNase I solution for 1 hour at 37°C. Cells were washed with cold PBS and stained the cells by Alexa-488 conjugated anti-BrdU solution for 1 hour. Then, cells were washed twice with cold PBS and treated with Hoechst 33342. Fluorescence from Alexa 488-BrdU and Hoechst33342 was observed with a fluorescent microscope (Nikon, Japan). Alexa 488-BrdU positive cells were counted by ImageJ software.

**Real-time qPCR**

Total RNAs was isolated using RNAiso Plus (Takara Biomedical, Japan) and cDNA was synthesized with cDNA kit (Intron, Korea). Real-time qPCR was carried out with Quantstudio 1 (Thermo Fisher Scientific, USA) and the following conditions were used: pre-incubation at 50 °C for 2 min, an initial melt at 95 °C for 10 min, and 40 cycles of amplification (95 °C for 15 s, 60 °C for 60 s) with 10 pmole of each primer. The primers used were described in Table S1. The data were normalized to GAPDH expression and the mRNA expression was compared according to the 2−ΔΔCT method.

**Immunostaining**

Treated cells were washed with PBS and fixed using 4% PFA and incubated for 10 min. 0.2% Triton X-100 (Sigma, USA) was added and incubated for 5 minutes and washed three times with 2% BSA in PBS. After that, 2% BSA was added and incubated for 60 minutes at room temperature. Then, the primary antibody in 2% BSA was added and incubated overnight at 4 °C. Cells were washed three times with PBS, treated with appropriate secondary antibody (Invitrogen, USA) and incubated for 1 hour at room temperature. After washing with PBS, the nuclei were stained using Hoechst (Thermo Fisher Scientific, USA), and cells were visualized with a fluorescent microscope (Nikon, Japan).

**Paracrine effect**

After LIPUS simulation on day 3, conditioned medium (CM) of early or late HS68 cells were collected and filtered through 0.22 μm filter. CM was diluted with DMEM 1:1 ratio and early or late HS68 cells were treated with the diluted CM for 3 days. After treatment, SA-β-gal activity and cell growth rate were analyzed.

**Western blot analysis**

Treated cells were rinsed twice with cold PBS and lysed in RIPA buffer for 20 min on ice. Lysates were cleared by centrifugation at 15,000 rpm for 30 min at 4°C. Protein concentration was measured by BCA Protein Assay kit (Thermo Fisher Scientific, USA). Equal amounts of total protein were subjected to SDS-PAGE and separated proteins were transferred to PVDF membranes. Membranes were blocked with 5 % skim milk at room temperature for 1 h, and then incubated with primary antibodies diluted in 5 % BSA/TBS-Tween 20 at 4 °C overnight. After washing, membranes were incubated with the appropriate HRP-conjugated secondary antibodies for 2 h at room temperature. Membranes were visualized by enhanced chemiluminescence (ATTO KOREA, Korea).

**Cell migration assay**

Cell migration assays were performed using transwell migration assay (#353097, Corning Inc, USA). Macrophage cells (Mo, M1 or M2) were seeded at a density of 5 × 10^4^ cells onto the upper chambers with RPMI containing 0.5 % FBS, and the upper chambers were placed into the lower chambers of 24-well culture plate containing supernatant obtained from treated HS68 cells. Following incubation for 8 h, migrated cells on the lower surface of the filter were fixed with 4% paraformaldehyde and stained with 0.2% crystal violet for 15 min at room temperature. Stained cells were observed under a light microscope (Nikon, Japan) and counted in at least five different representative microscopic fields (×100) per filter.

**Human cytokine array**

Supernatant obtained from treated cells culture media was used for human cytokine array (#ARY005B, R&D Systems, USA) according to the manufacturer’s protocol. In brief, ultrasound treated HS68 cells were incubated for 3 days. Then culture media was centrifuged at 14,000 rpm for 10 min to eliminate any debris. Supernatant was diluted and mixed with a biotinylated detection antibody cocktail. The mixture was then incubated overnight with an array membrane at 4 °C. Membrane was washed three times and incubated with streptavidin-horseradish peroxidase antibody for 30 min at RT and then detected by chemiluminescence (ATTO KOREA, Korea). The obtained immunoblot images were analyzed using ImageJ software.

**Measurement of ROS production**

For the measurement of extracellular ROS by LIPUS stimulation, TA (2 mM in PBS) was added to the confocal dish. LIPUS stimulated solution was transferred to 96 well plate, and the fluorescence from HTA, reaction product of TA with ROS was measured by fluorescence microplate reader Appliskan (Thermo Fisher Scientific, USA) with excitation/emission filter sets of 326/16 and 460/20 nm. The fluorescence from HTA was also measured to produce a calibration curve.

The intracellular ROS levels were measured using ROS Detection Cell-Based Assay Kit (Cayman Chemical, USA). Cells were harvested and washed with cell-based assay buffer. The cells were incubated with 5 μM dihydroethidium (DHE) for 30 min at 37°C in the dark and washed twice with cold PBS. Then, fluorescence intensity from DHE was analyzed using a flow cytometer (CytoFLEX, Beckman, USA).

**Phagocytosis assay**

Phagocytosis assay was performed as described previously.(Nam et al., 2019) LIUPS stimulated HS68 cells or non-treated HS68 cells were cultured for 3 days, and the supernatant was separately collected at day 3. Treated or non-treated HS68 cells were labeled with Vybrant^TM^ DiD cell labeling solution (DiD 1 μM, Thermo Fisher Scientific, USA) for 30 min at RT and then washed with PBS three times. Then, 4.0 × 10^4^ of DiD labeled HS68 cells were seeded on 8.0 × 10^4^ of M1 polarized macrophage in the supernatant collected from the same cells as stated above and co-cultured for 24 h at 37°C. The mixture of HS68 and M1 cells were washed with cold PBS and stained with PE-Cy7-CD45 for 30 min at RT. After staining, the mixture of DiD labeled-HS68 and PE-Cy7-CD45 labeled-M1 macrophage were analyzed flow cytometry (CytoFLEX, Beckman, USA). Both PE-Cy7-CD45 and DiD positive cells were quantified.

**Membrane permeability assay (sonoporation)**

HS68 cells (1 × 10^4^ cells /cm^2^) were seeded on a poly-L-lysine (0.1 mg/mL) coated 35 mm-sized confocal dish (SPL, Korea) and either FITC-BSA (5 μM) or mCherry plasmid (pCMV-mCherry from addgene, 5ug) were added to the media. After LIPUS stimulation, treated cells were washed with PBS, detached from the confocal dish, fixed with 4% PFA for 10 min at 4°C and then analyzed by flow cytometry (CytoFLEX, Beckman, USA). In case with mCherry treated cells, the fluorescence from mCherry was exmined under a fluorescence microscope (Nikon, Japan).

**Mice model for UVA induced senescence and LIPUS stimulation**

All animal experiments were performed under the protocol approved by the institutional animal care and use committee (KIST-IACUC-2022-035-1). Six-week-old male SKH1-hr mice were purchased from Orient Bio Inc. (Gyeonggi-do, Korea). Five or six mice were housed in a cage that had a 12 h light/dark cycle. Temperature in the animal facility was maintained at 22 ± 2 °C, and humidity at 55 ± 5%. All mice were acclimatized for 6 days ahead of the experiments and provided with ad libitum access to food and water.

For the UVA induced skin senescence model, the mice were randomly assigned to four groups, with five mice allocated to each group. Group 1 represents the untreated control group, Group 2 received LIPUS treatment only, Group 3 received UVA irradiation only and Group 4 received UVA irradiation followed by LIPUS treatment. In the experiment, mice were anesthetized using a combination of alfaxalone (100 mg/kg; Jurox) and xylazine (20 mg/kg; elanco korea) before the experiments. UVA irradiation was administered daily at a dose of 10 J/cm^2^ on the dorsal skin, resulting in a total cumulative dose of 100 J/cm^2^. After 5 days of UVA irradiation, there was a 2-day break, followed by another 5 days of irradiation. After the completion of UVA irradiation, a 10-day resting period was provided.

For the LIPUS exposure on the dorsal skin of the mice, the transducer was positioned above the holder (Figure 6). Ultrasound transmission gel (Sapina Inc.) was used to ensure full contact to the dorsal skin, and LIPUS treatment was administered for 30 minutes each day, consecutively for 5 days. After a resting period of 10 days following the completion of LIPUS treatment, the mice were sacrificed, and dorsal skin samples were used for analysis.

**Histological analysis of the mouse skin**

Histological analysis was performed by Labcore company (Seoul, Korea). Briefly, Paraffin embedded mouse skin samples were sliced into 4 μm sections, stained with hematoxylin and eosin (H&E) solution. For immunohistochemistry evaluation, samples were incubated overnight in a water bath set to 60 °C and cooled down for epitope retrieval and antibody staining. Samples were incubated with appropriate antibodies (described in the Materials section) at 4 °C overnight, and then ImmPRESS ® HRP antibody detection kit (Vector Lab, USA) was used for detection. Samples were counterstained with Mayer’s hematoxylin, and visualized with an optical scanner (Motic EasyScan, Motic Scientific, USA). For immunofluorescence, anti-rat IgG (H+L), (Alexa Fluor® 488 Conjugate, Thermo Fisher Scientific, USA) and anti-Rabbit IgG (H+L), (Alexa Fluor® 594 Conjugate, Thermo Fisher Scientific, USA) were used to detect p53 and CD11b, respectively. Nucleus was staining with Fluoroshield ^TM^ with DAPI (Sigma Aldrich, USA). Slides were visualized under a fluorescence microscope (Olympus BX53, Olympus, Japan). The images were quantified with ImageJ software.

**Statistics**

All the measurement data is from at least 3 independent replicative experiments, and quantitative data are expressed as the means ± standard error of mean (SEM). Statistical analyses with Student’s t-test and ANOVA were performed using Origin Pro 9.0 software.

**
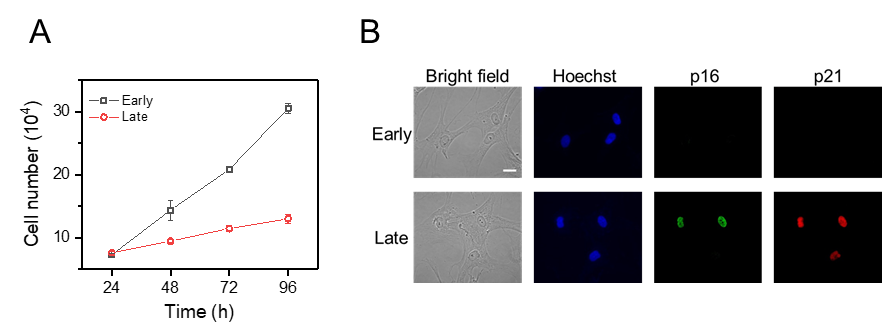
**

**Figure S1. Characterization of senescent HS68 cells.** (A) Proliferation rate of early and late HS68 cells. Cells were counted as described in Materials and Methods. (A) Immunofluorescent images of early and late HS68 cells stained with Hoechst, p16, and p21. Scale bar, 100 μm.

**
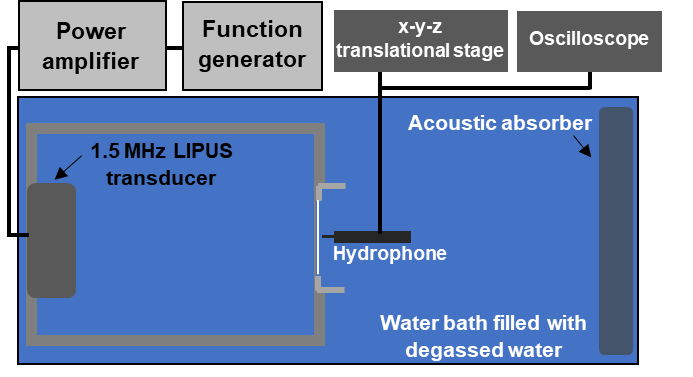
**

| Input voltage (mVpp) | Peak Pressure (kPa) | Ispta (mW/cm^2^) | Input voltage (mVpp) | Peak Pressure (kPa) | I_spta_ (mW/cm^2^) |
| --- | --- | --- | --- | --- | --- |
| 0 | 0 | 0 | 137 | 122 | 200 |
| 28 | 23.6 | 7.5 | 170 | 147.1 | 300 |
| 39 | 33.6 | 15 | 190 | 172.3 | 400 |
| 53 | 47.5 | 30 | 235 | 211 | 600 |
| 76 | 67 | 60 | 280 | 243.5 | 800 |
| 106 | 94.4 | 120 | 380 | 334 | 1600 |

Figure S2. Low intensity pulsed ultrasound (LIPUS) experimental set up used for calibrating the 1.5 MHZ LIPUS transducer. Peak pressure was converted to spatial average temporal average intensity, I_spta_, as shown in the table.

**
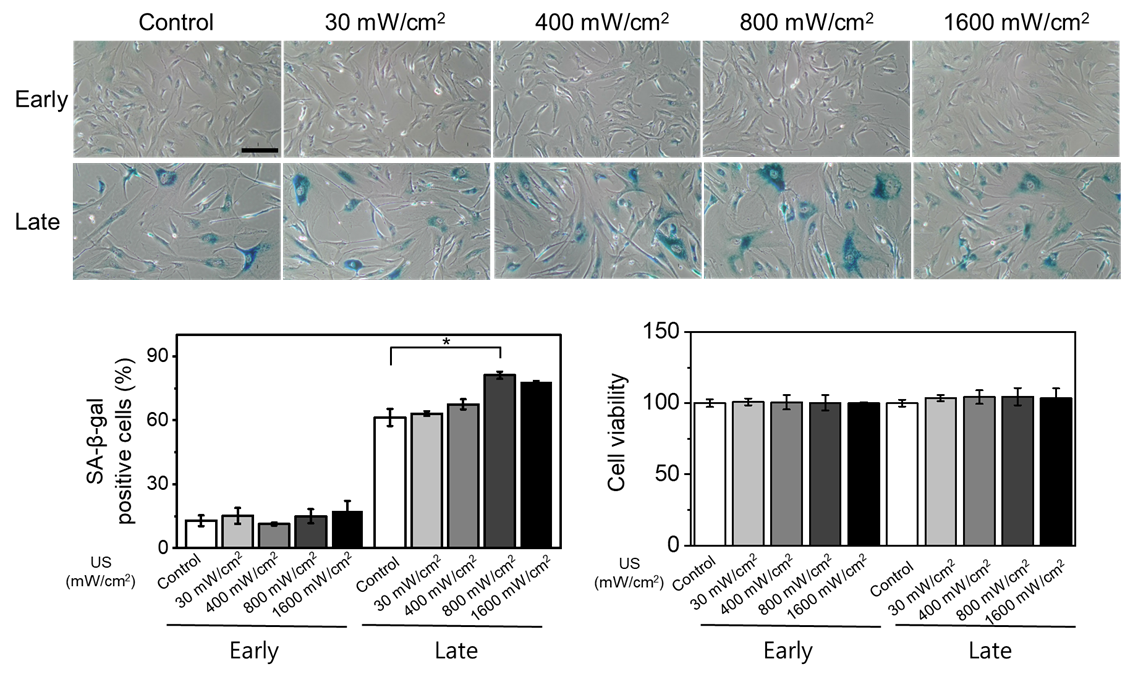
**

**Figure S3. Optimization of ultrasound stimulation I_spta_.** SA β-gal staining images of early and late HS68 cells, and cell viability measured after 3 days of LIPUS treatment. I_spta_ was increased from 0 to 1600 mW/cm^2^. Scale bar, 100 µm.


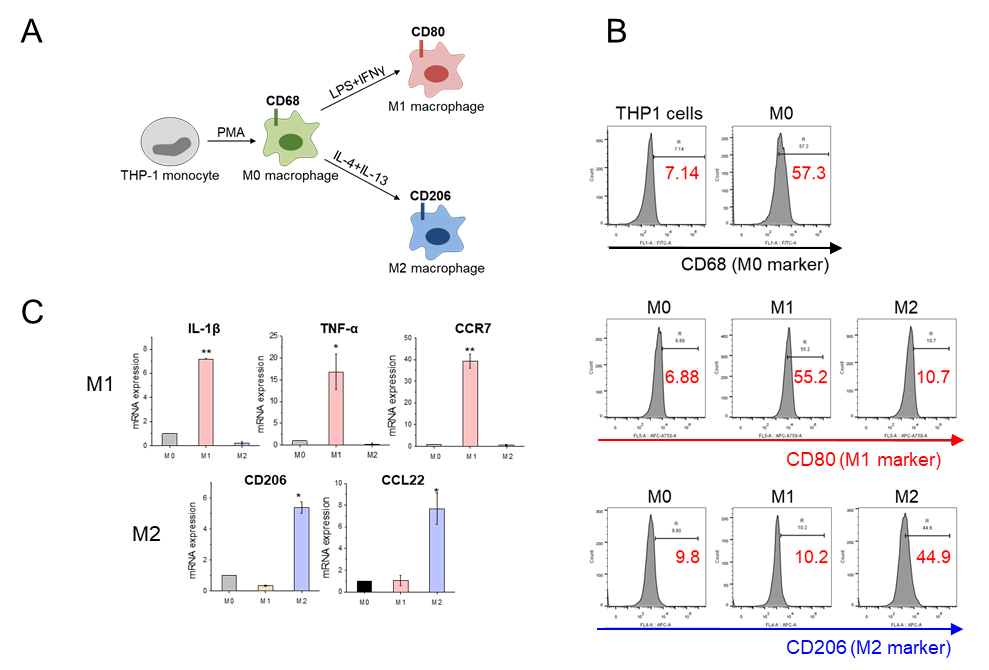


**Figure S4. Validation of macrophage polarization.** (A) M0 macrophages, differentiated from THP-1 cells, were polarized into M1 and M2 phenotypes as described in Materials and Methods. The degree of polarization was validated by (B) surface marker expression (M0: CD68, M1: CD80, and M2: CD206) using FACS analysis and (C) specific cytokine expression (M1: IL-1β, TNFα, and CCR7; M2: CCL22 and CD206) using real-time qPCR.


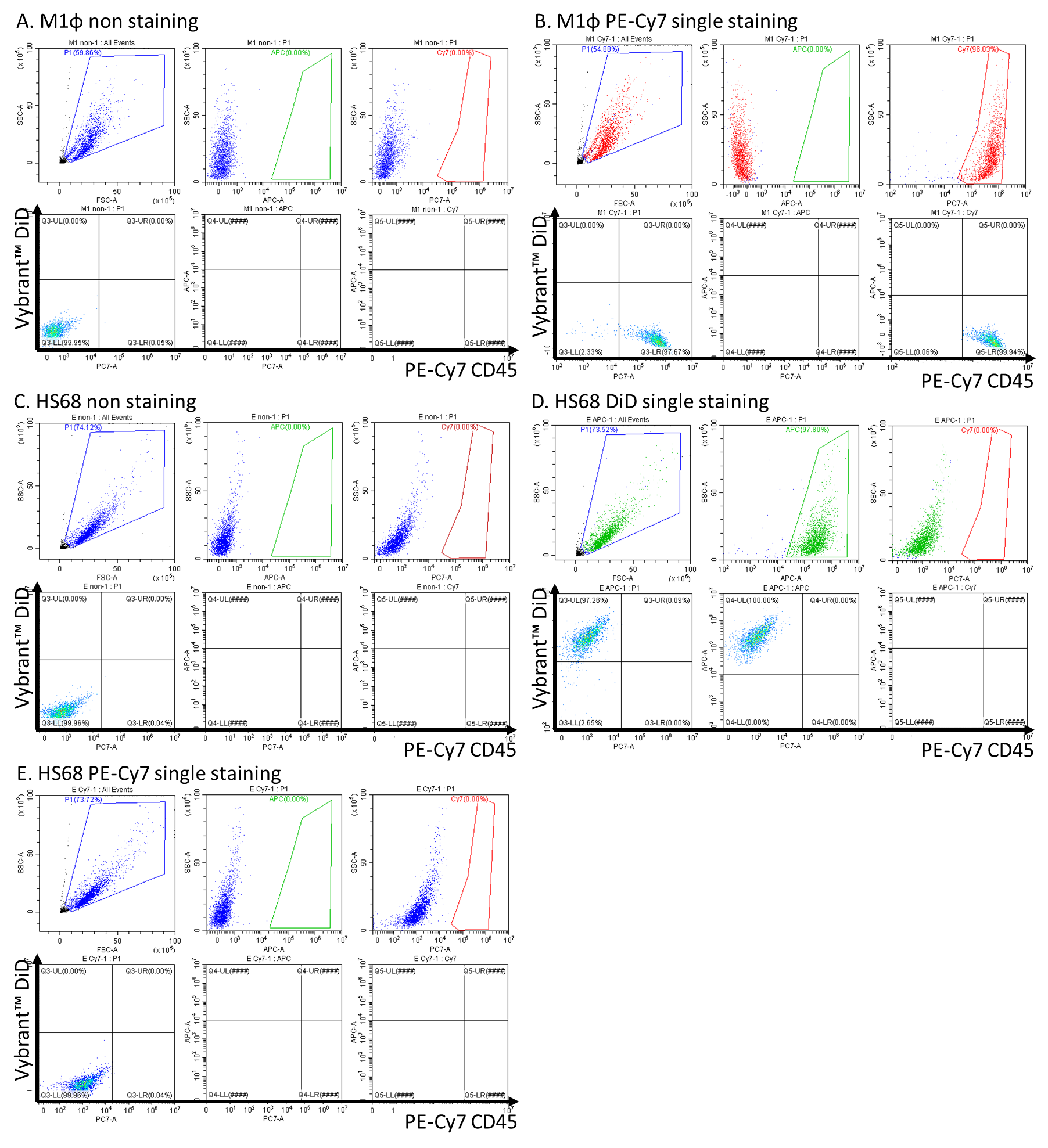


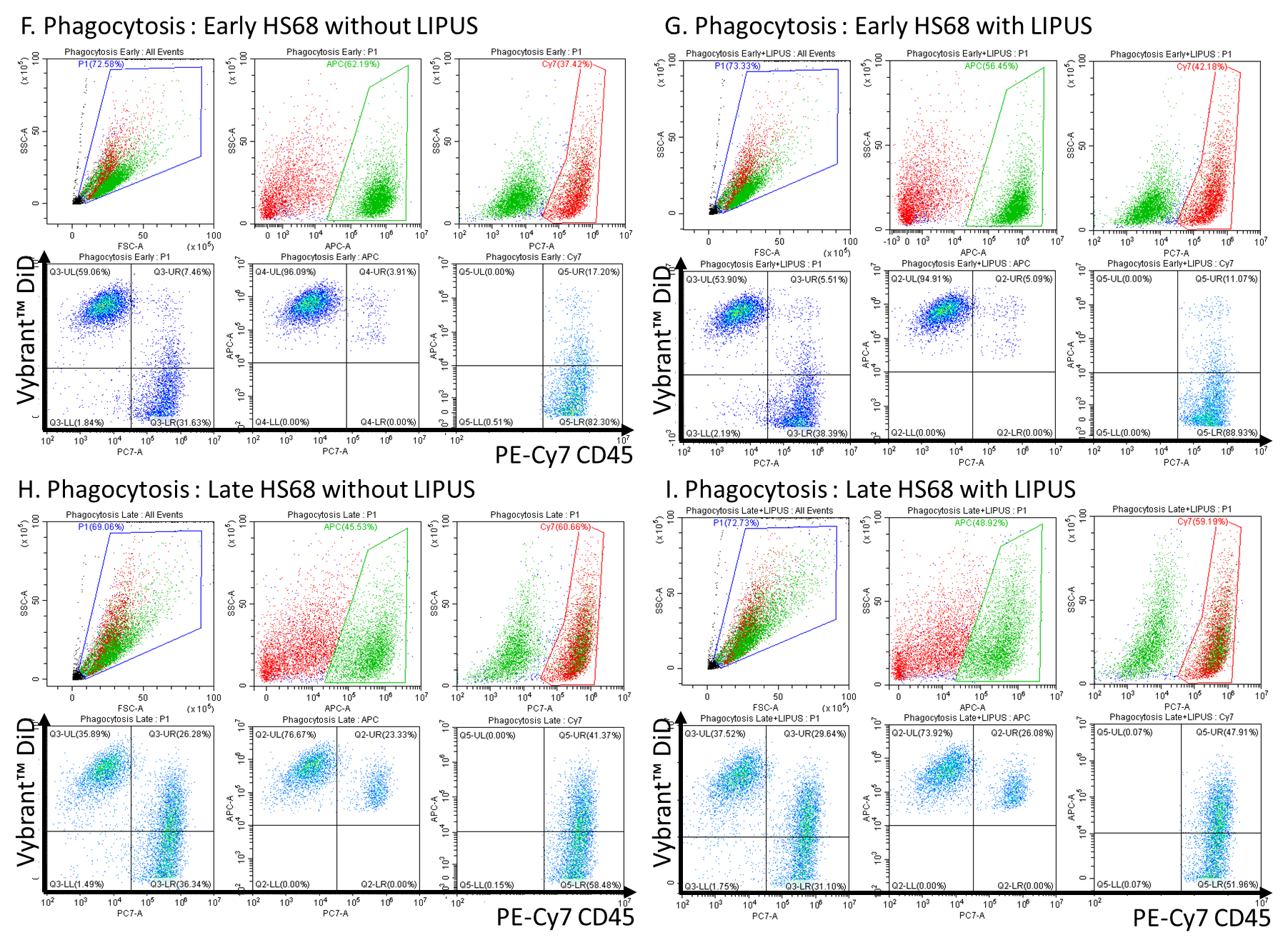


**Figure S5. FACS analysis of phagocytosis assay.** (A) FACS histogram of non-stained M1 macrophage cells. (B) FACS histogram of PE-Cy7 labeled CD45 single stained M1 macrophage cells. (C) FACS histogram of non-stained HS68 cells. (D) FACS histogram of DiD single stained HS68 cells. (E) FACS histogram of PE-Cy7 labeled CD45 single stained HS68 cells. (F) FACS histogram of phagocytosis assay: Early HS68 without LIPUS treatment. (G) FACS histogram of phagocytosis assay: Early HS68 with LIPUS treatment. (H) FACS histogram of phagocytosis assay: Late HS68 without LIPUS treatment. (I) FACS histogram of phagocytosis assay: Late HS68 with LIPUS treatment.

**
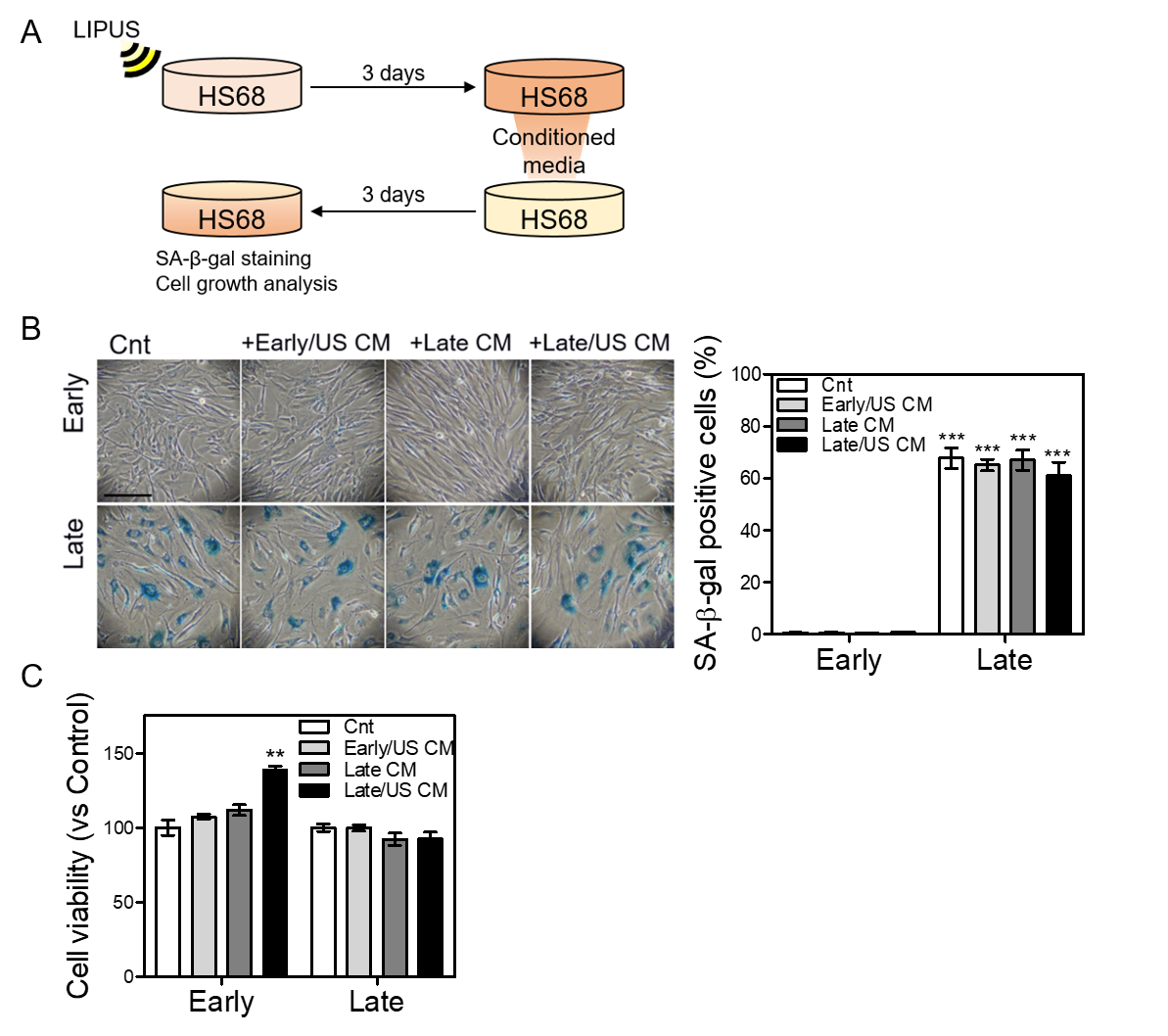
**

**Figure S6. Paracrine effects of LIPUS stimulation.** (A) The schematic diagram of paracrine effects analysis. (B) SA-β-gal activity in early and late HS68 cells treated with the CM. (C) Cell viability of early and late HS68 cells treated with the CM. Data represent means ± SEM of three independent experiments. *** P < 0.01 and *** P < 0.001.*


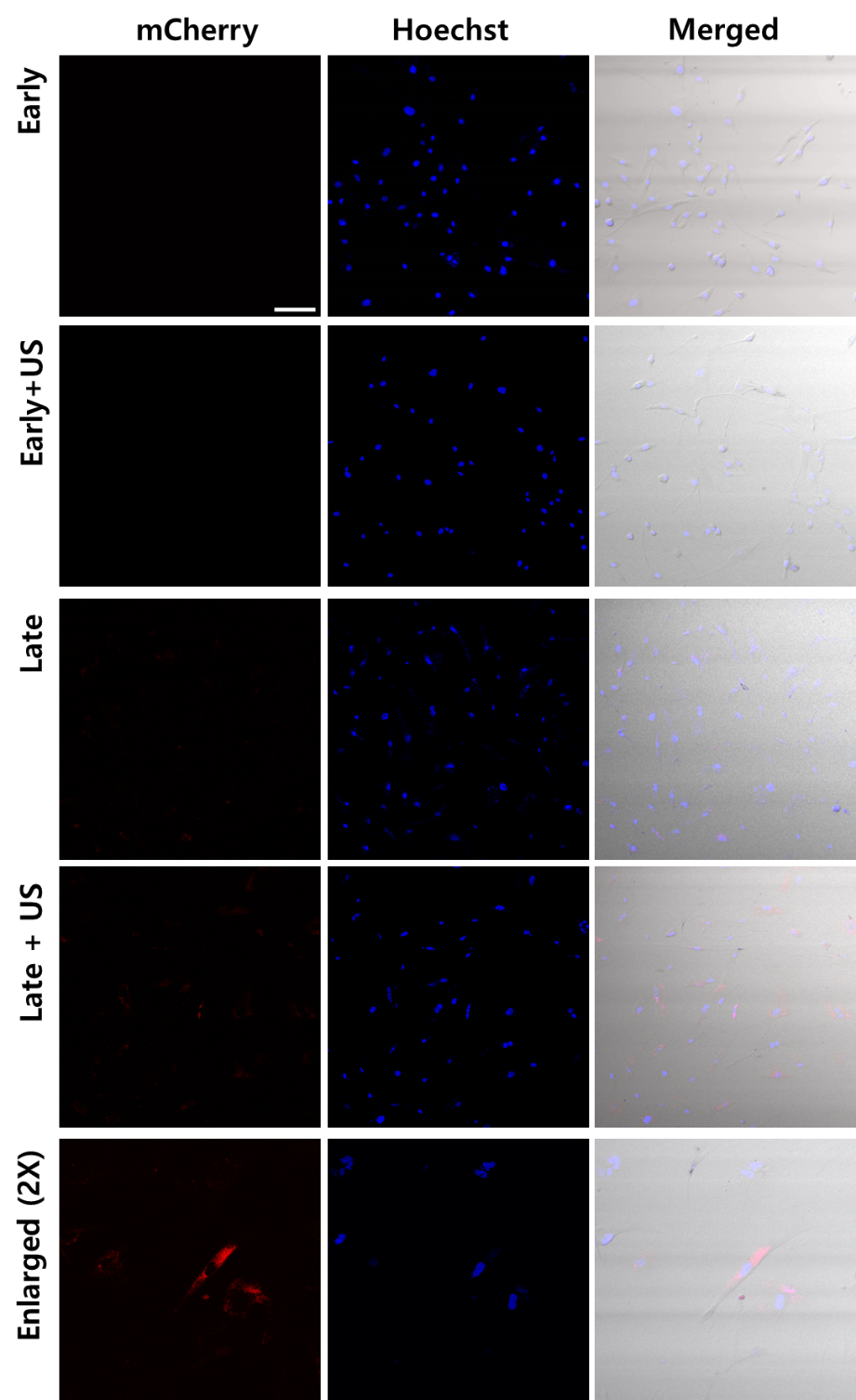


**Figure S7. Sonoporation effects of LIPUS stimulation.** Early and late HS68 cells treated with an mCherry plasmid (pCMV-mCheery). Treated cells were stimulated with or without LIPUS and mCherry fluorescence was examined after 24 h using fluorescence microscope. To confirm the cytoplasmic expression of mCherry protein, a separate enlarged (2X) image was taken.


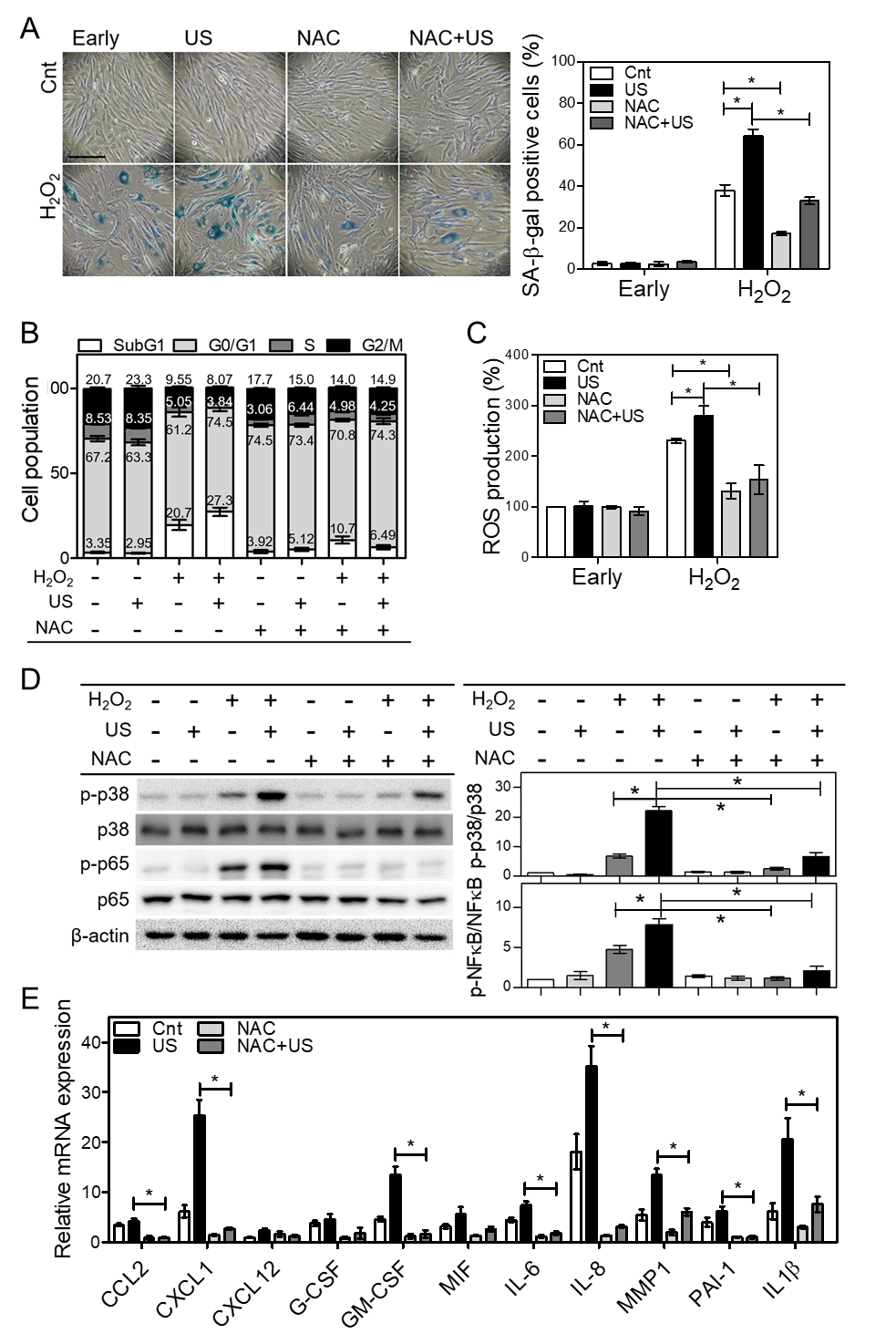


**Figure S8**. **LIPUS stimulation enhances the SASP via the ROS-dependent p38-NFκB pathway in oxidative stress (H_2_O_2_)-induced senescence model.** For oxidative stress induced senescence model, 100 μM of H_2_O_2_ treated twice for every 3 days in early HS68 cells. To evaluate the role of ROS in LIPUS stimulation, 10 mM of NAC was used prior to LIPUS stimulation. (A) SA-β-gal activity of early and H_2_O_2_ treated HS68 cells in the presence or absence of NAC. Cells stimulated with or without LIPUS were analyzed on Day 3. (B) Cell cycle analysis of early and H_2_O_2_ treated HS68 cells in the presence or absence of NAC. Cells stimulated with or without LIPUS were analyzed on Day 3. (C) ROS production in early and H_2_O_2_ treated HS68 cells in the presence or absence of NAC. Cells stimulated with or without LIPUS were analyzed on Day 3. (D) Western blot analysis of p-p38 and p-NF-κB. The protein expression levels of p-p38 and p-NF-κB were measured in early and H_2_O_2_ treated cells in the presence or absence of NAC. Cells stimulated with or without LIPUS were analyzed on Day 3. Data were normalized to the corresponding total protein levels. (E) Real-time qPCR analysis of SASP factors. Early and H_2_O_2_ treated cells were incubated with or without NAC. Cells stimulated with or without LIPUS were analyzed on Day 3. Data represent means ± SEM of three samples. ** P < 0.05.*

**
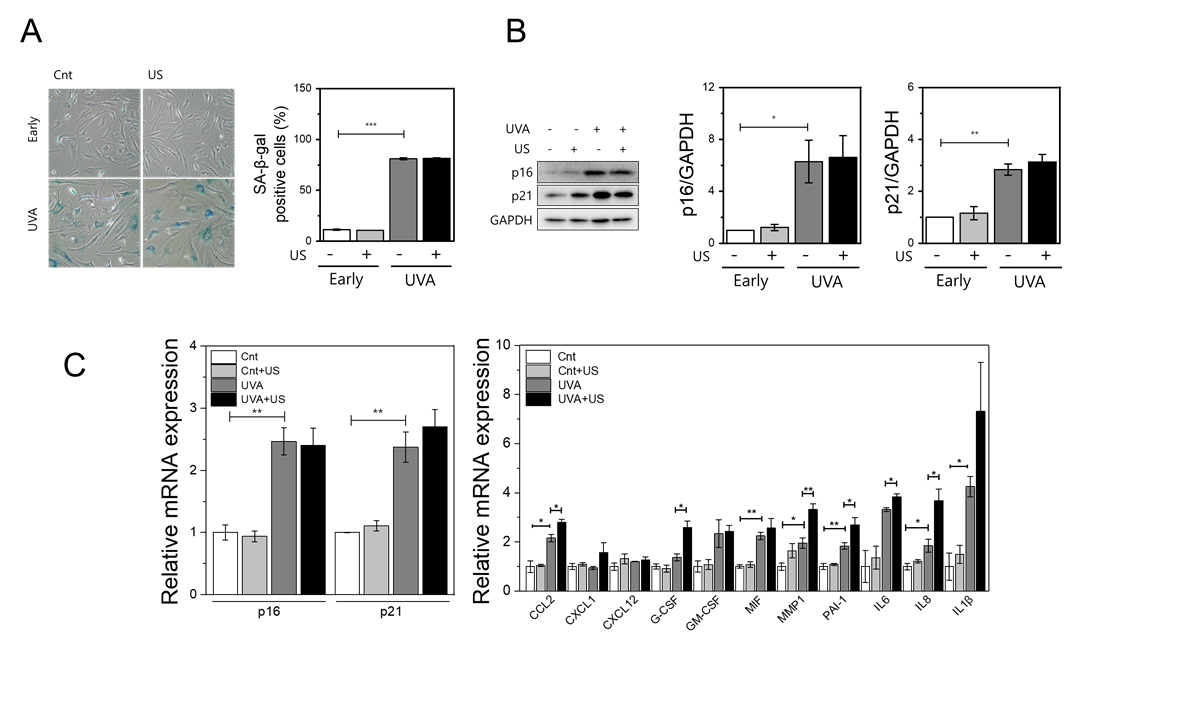
**

**Figure S9. LIPUS stimulation enhances the SA-β-gal activity and SASP factors in UVA induced senescent HS68 cells *in vitro.*** (A) SA-β-gal activity of early and UVA treated HS68 cells in the presence or absence of LIPUS stimulation. Cells stimulated with or without LIPUS were analyzed on Day 3. (B) Western blot analysis of p16 and p21. The protein expression levels of p16 and p21 were measured in early and UVA treated cells in the presence or absence of LIPUS stimulation. Cells stimulated with or without LIPUS were analyzed on Day 3. Data were normalized to the corresponding total protein levels. (C) Real-time qPCR analysis of p16, p2 and SASP factors. Early and UVA treated cells with or without LIPUS stimulation were analyzed on Day 3. Data represent means ± SEM of three samples. ** P < 0.05*, *** P < 0.01, and *** P < 0.001.* Although SA-β-gal activity in UVA-treated cells was not significantly upregulated following LIPUS stimulation, the upregulation of SASP factors was consistently observed.

**
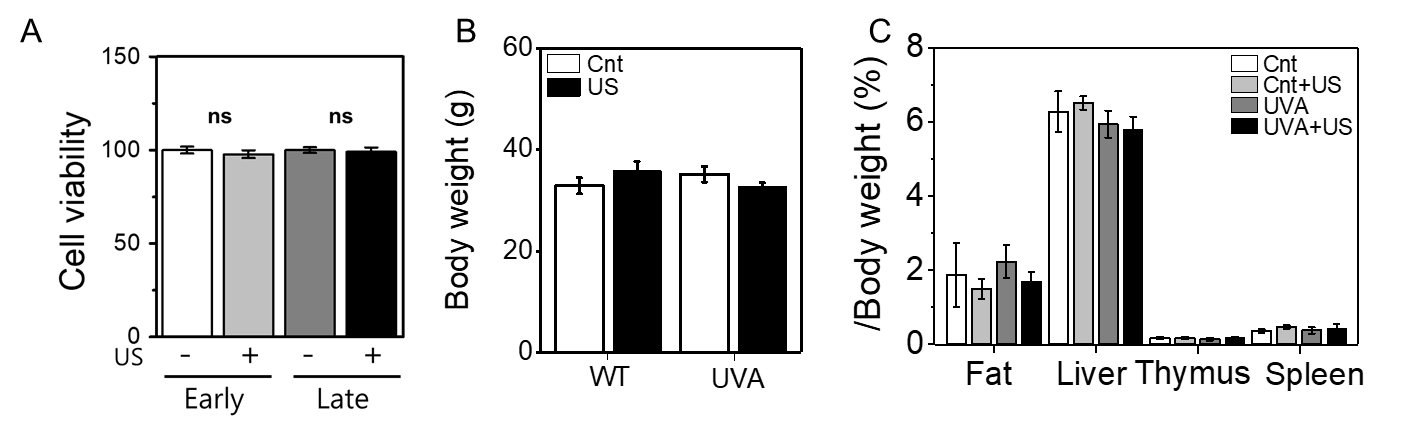
**

**Figure S10. Neither UVA nor** **LIPUS treatment influenced the cell viability *in vitro*, and weight of body and major organs *in vivo*.** (A) Cell viability after five days of consecutive LIPUS stimulation *in vitro* with HS68 cells. Data represent means ± SEM of three samples. (B) Body weight of the mice after UVA or LIPUS treatment. (C) Organ weight relative to body weight of the mice after UVA or LIPUS treatment.

**
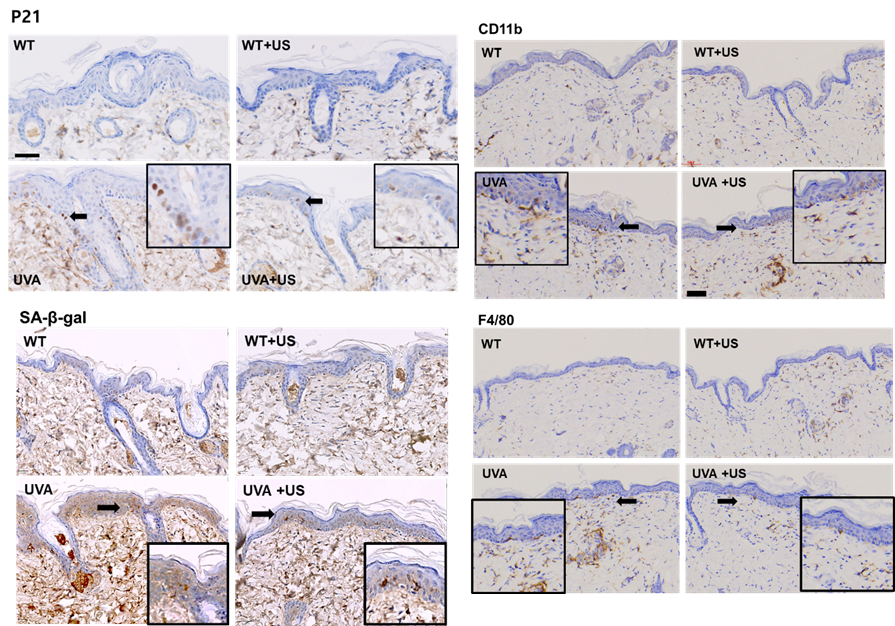
**

**Fig. S11.** **Enlarged images of p21, SA-β-gal, CD11b and F4/80 immunohistochemistry (IHC) staining from Fig. 6E and 6G.** Scale bar, 40 μm for p21 and SA-β-gal. Scale bar, 60 μm for CD11b and F4/80.

**
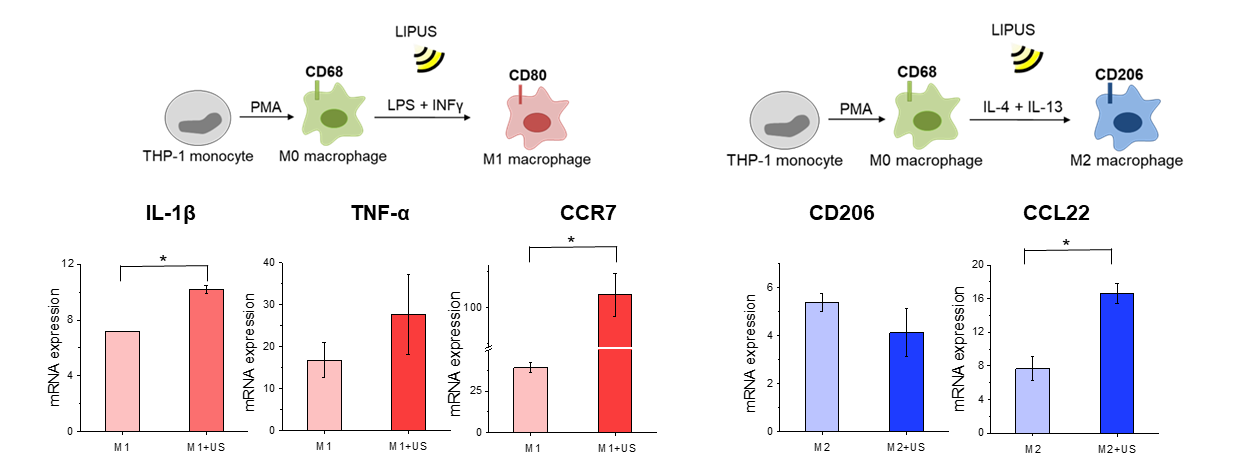
**

**Figure S12. LIPUS treatment affects cytokine production of M1 and M2 macrophages.** The THP-1 cells were activated by PMA, and then polarized to either M1 or M2 phenotypes by appropriate cytokines. The mRNA expression of M1- or M2- specific cytokines in the absence or presence of LIPUS stimulation was analyzed by Real-time qPCR.

**Table S1.** qPCR primer sequences for human and mouse

**Human PCR primer**

| Gene | Sequence (5’→3’) |
| --- | --- |
| Rb | Forward: GGAAGCAACCCTCCTAAACC  Reverse: TTTCTGCTTTTGCATTCGTG |
| ATM | Forward: ATCTGCTGCCGTCAACTAGAA  Reverse: GATCTCGAATCAGGCGCTTAAA |
| ATR | Forward: GGCCAAAGGCAGTTGTATTGA  Reverse: GTGAGTACCCCAAAAATAGCAGG |
| p53 | Forward: CAGCACATGACGGAGGTTGT  Reverse: TCATCCAAATACTCCACACGC |
| p16 | Forward: GATCCAGGTGGGTAGAAGGTC  Reverse: CCCCTGCAAACTTCGTCCT |
| p21 | Forward: AGGTGGACCTGGAGACTCTCAG  Reverse: TCCTCTTGGAGAAGATCAGCCG |
| p27 | Forward: ATGTCAAACGTGCGAGTGTC  Reverse: TCTCTGCAGTGCTTCTCCAA |
| Cyclin D1 | Forward: ATGAGCACTGAAAGCATGATCC  Reverse: GAGGGCTGATTAGAGAGAGGTC |
| Cyclin E | Forward: CAGATTGCAGAGCTGTTGGA  Reverse: TCCCCGTCTCCCTTATAACC |
| Cyclin A | Forward: TTATTGCTGGAGCTGCCTTT  Reverse: CTCTGGTGGGTTGAGGAGAG |
| Cyclin B | Forward: CGGGAAGTCACTGGAAACAT  Reverse: AAACATGGCAGTGACACCAA |
| CCL2 | Forward: GAGAGGCTGAGACTAACCCAGA  Reverse: ATCACAGCTTCTTTGGGACACT |
| CCL3 | Forward: GGCTCTCTGCAACCAGTTCT  Reverse: TGAAATTCTGTGGAATCTGCC |
| CXCL1 | Forward: GAAAGCTTGCCTCAATCCTG  Reverse: CTTCCTCCTCCCTTCTGGTC |
| CXCL12 | Forward: ATGCCCATGCCGATTCTTCG  Reverse: GCCGGGCTACAATCTGAAGG |
| G-CSF | Forward: TCCCCATCCCATGTATTTATCT  Reverse: AACTCAGAAATGCAGGGAAGGA |
| GM-CSF | Forward: CACTGCTGCTGAGATGAATGAAA  Reverse: GTCTGTAGGCAGGTCGGCTC |
| MIF | Forward: GAACAACTCCACCTTCGCCT  Reverse: CCGTTTATTTCTCCCCACCA |
| IL6 | Forward: CCACACAGACAGCCACTCACC  Reverse: CTACATTTGCCGAAGAGCCCTC |
| IL8 | Forward: CTCTCTTGGCAGCCTTCCTGATT  Reverse: AACTTCTCCACAACCCTCTGCAC |
| MMP1 | Forward: AGCTAGCTCAGGATGACATTGATG  Reverse: GCCGATGGGCTGGACAG |
| PAI-1 | Forward: ACCTGGGAATGACCGACATGT  Reverse: CTCTCGTTCACCTCGATCTTCACT |
| IL-1β | Forward: CTGTCCTGCGTGTTGAAAGA  Reverse: TTGGGTAATTTTTGGGATCTACA |
| TGF-β1 | Forward: AGGGCTACCATGCCAACTTCT  Reverse: CCGGGTTATGCTGGTTGTACA |
| TNFα | Forward: ATGAGCACTGAAAGCATGATCC  Reverse: GAGGGCTGATTAGAGAGAGGTC |
| NOX4 | Forward: GCAGGAGAACCAGGAGATTG  Reverse: CACTGAGAAGTTGAGGGCATT |
| CCL22 | Forward: ATTACGTCCGTTACCGTCTG  Reverse: TAGGCTCTTCATTGGCTCAG |
| CD206 | Forward: GGACGTGGCTGTGGATAAAT  Reverse: ACCCAGAAGACGCATGTAAAG |
| CCR7 | Forward: CAGACAGGGGTAGTGCGAGGC  Reverse: CCAGCACGCTTTTCATTG |
| GAPDH | Forward: GGAGCGAGATCCCTCCAAAAT  Reverse: GGCTGTTGTCATACTTCTCATGG |

**Mouse PCR primer**

| p21-m | Forward: CCTGGTGATGTCCGACCTG  Reverse: CCATGAGCGCATCGCAATC |
| --- | --- |
| p53-m | Forward: TCATCCCTCCCCTTTTCTGTC  Reverse: ATGGCGGGAAGTAGACTGGC |
| CCL2-m | Forward: GTCTGTGCTGACCCCAAGAAG  Reverse: TGGTTCCGATCCAGGTTTTTA |
| CCL3-m | Forward: GCGGCTGATGATTGGACAA  Reverse: ATCTCCAGCTCGAGCAATGG |
| CXCL1-m | Forward: GACTCCAGCCACATCCAAC  Reverse: TGACAGCGCAGCTCATTG |
| CXCL12-m | Forward: CGCCAAGGTCGTCGCCG  Reverse: TTGGCTCTGGCGATGTGGC |
| G-CSF-m | Forward: CGTTCCCCTGGTCAGTGTC  Reverse: CCGCTGGCCTGGATCTTC |
| GM-CSF-m | Forward: GGCTCACTGGCCCCATGTAT  Reverse: GAGTACTGGGCTCACTGCAA |
| MIF-m | Forward: ACAGCATCGGCAAGATCG  Reverse: GGCCACACAGCAGCTTACT |
| IL6-m | Forward: ACCACGGCCTTCCCTACTTC  Reverse: TTGGGAGTGGTATCCTCTGTGA |
| IL8-m | Forward: GCTGTGACCCTCTCTGTGAAG  Reverse: CAAACTCCATCTTGTTGTGTC |
| PAI-1-m | Forward: CTCCAAGGGGCAACGGATAG  Reverse: AAGCAAGCTGTGTCAAGGGA |
| IL-1β-m | Forward: TCAGGCAGGCAGTATCACTCA  Reverse: CACGGGAAAGACACAGGTAGCT |
| TNFα-m | Forward: TCCCAGGTTCTCTTCAAGGGA  Reverse: GGTGAGGAGCACGTAGTCGG |
| β-actin | Forward: GATGTATGAAGGCTTTGGTC  Reverse: TGTGCACTTTTATTGGTCTC |

R**eferences**

Chen, J.-H., Ozanne, S. E., & Hales, C. N. (2007). *Methods of Induction of Cellular Senescence Using Oxidative Stress* (T. O. Tollefsbol Ed. Vol. 371). New Jersey: Humana Press Inc.

Lozano-Gerona, J., & Garcia-Otin, A. L. (2018). ImageJ-based semiautomatic method to analyze senescence in cell culture. *Anal Biochem, 543*, 30-32. doi:10.1016/j.ab.2017.11.020

Nam, G.-H., Hong, Y., Choi, Y., Kim, G. B., Kim, Y. K., Yang, Y., & Kim, I.-S. (2019). An optimized protocol to determine the engulfment of cancer cells by phagocytes using flow cytometry and fluorescence microscopy. *Journal of Immunological Methods, 470*, 27-32. doi:10.1016/j.jim.2019.04.007
